# Supplementary material for: The G9a-TRIM21 axis exacerbates diabetic renal ischemia-reperfusion injury by inducing methylation-dependent ubiquitination and degradation of FoxO3a to promote oxidative stress and pyroptosis
Source: Redox Biol. 2025 Dec 8;89:103964. doi: 10.1016/j.redox.2025.103964 (PMC12750793; doi:10.1016/j.redox.2025.103964)

**Abstract**

**Introduction**

Diabetic renal ischemia-reperfusion injury (RIRI) is a severe surgical complication with particularly poor outcomes in diabetic patients. The histone methyltransferase G9a has been implicated in various pathological processes, but its role in diabetic RIRI remains unclear. Emerging evidence suggests that non-histone protein methylation may play crucial roles in cellular responses to ischemic injury.

**Objectives**

This study aimed to investigate the functional role of G9a in diabetic RIRI and elucidate its molecular mechanisms, with particular focus on its regulation of FoxO3a stability, oxidative stress and cellular pyroptosis.

**Methods**

We established diabetic RIRI models using G9a conditional knockout mice and HK-2 cells under high glucose conditions. Renal function was assessed through serum creatinine and BUN measurements. Histopathological evaluation and molecular analyses were performed to examine tissue damage and pyroptosis markers. Mechanistic studies included mass spectrometry identification of G9a-interacting proteins, co-immunoprecipitation to verify protein interactions, and ubiquitination assays to characterize post-translational modifications. Site-directed mutagenesis was employed to identify critical residues in FoxO3a regulation.

**Results**

G9a expression was significantly upregulated in diabetic RIRI models. Genetic ablation of G9a attenuated renal injury and reduced oxidative stress and pyroptosis in both in vivo and in vitro models. Mechanistically, G9a directly interacted with and methylated FoxO3a at lysine 262, which facilitated its recognition by the E3 ubiquitin ligase TRIM21. TRIM21 subsequently mediated K48-linked polyubiquitination of FoxO3a at lysine 176, leading to proteasomal degradation. This G9a-mediated FoxO3a degradation promoted oxidative stress, NLRP3 inflammasome activation and pyroptosis. Importantly, pharmacological inhibition of G9a with BIX-01294 or FoxO3a overexpression significantly ameliorated diabetic RIRI.

**Conclusion**

Our study reveals a novel G9a-TRIM21-FoxO3a regulatory axis in diabetic RIRI, where G9a-mediated methylation licenses FoxO3a ubiquitination and degradation, thereby promoting pyroptosis and oxidative stress. These findings identify G9a as a potential therapeutic target for preventing or treating diabetic kidney ischemia-reperfusion injury.

**Keywords** G9a; FoxO3a; Ischemia reperfusion injury; TRIM21; Oxidative stress

**Graphic abstract**


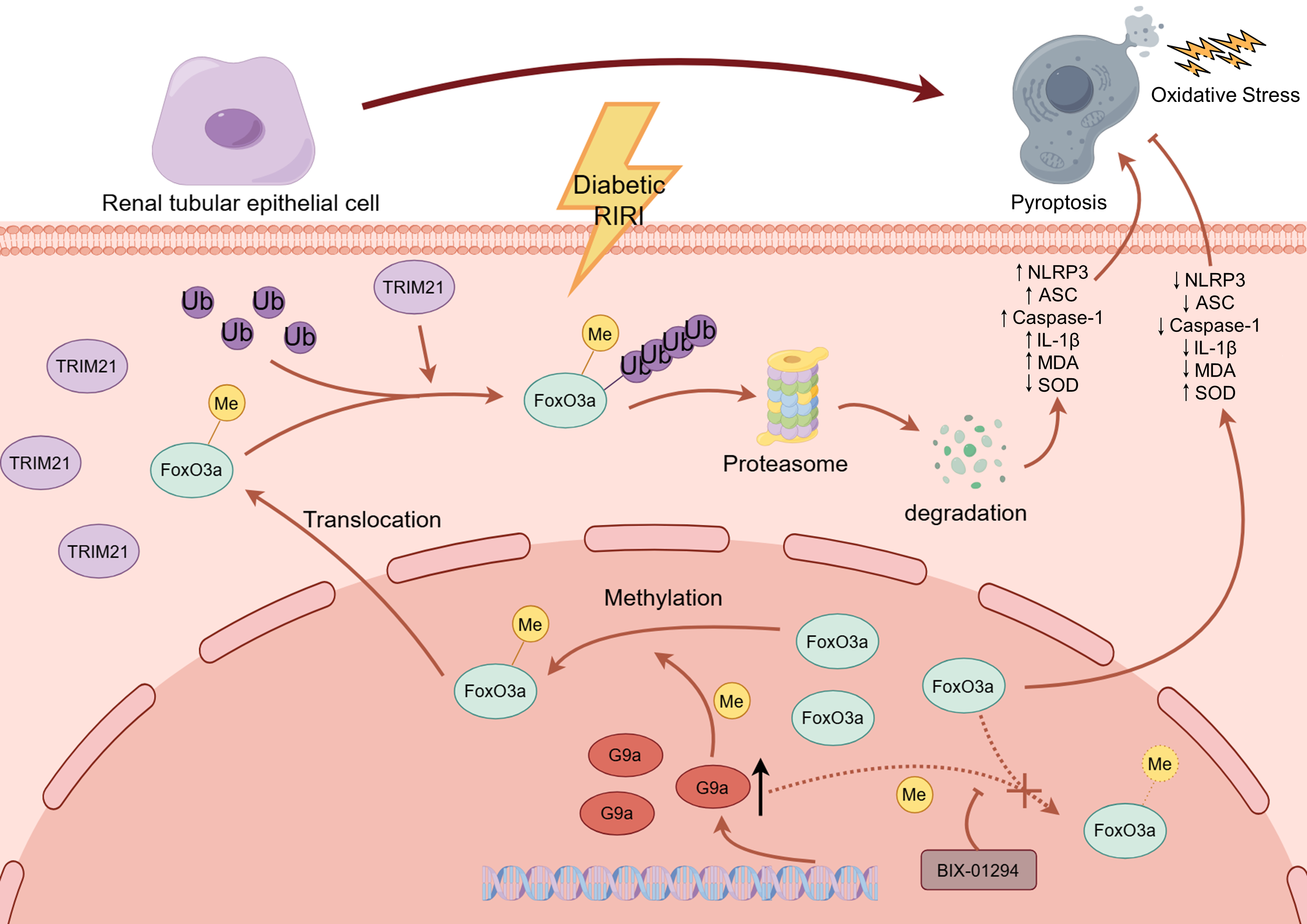


**Highlight**

- G9a is the key driver of oxidative stress and pyroptosis and regulates the degradation and translocation of FoxO3a by methylation in diabetic RIRI.
- The K-48 linked ubiquitination of FoxO3a is mediated by TRIM21 in diabetic RIRI.
- The methylation of FoxO3a is prerequisite for following ubiquitination in diabetic RIRI.
- The inhibitor of G9a methyltransferase activity shows significant renal protective effect in diabetic RIRI.

**1 Introduction**

Acute kidney injury (AKI) manifests as a rapid decline in kidney function, accumulation of metabolic toxins, and concurrent multi-organ dysfunction [1]. It remains a global health burden with high incidence and mortality [2, 3]. Renal ischemia-reperfusion injury (RIRI) is a primary cause of AKI, arising from conditions such as shock, circulatory failure, transplantation, and surgical procedures [4]. Its pathological hallmarks include tubular necrosis, cast formation, and elevated biomarkers including Kidney Injury Molecule-1 (KIM1) and Neutrophil Gelatinase-Associated Lipocalin (NGAL) [4]. Critically, diabetes mellitus (DM) significantly increases susceptibility to RIRI [5, 6], yet effective therapeutic strategies remain lacking, highlighting the urgent need for novel interventions.

The functional diversity of proteins is extensively regulated through post-translational modifications (PTMs), which serve as key mechanisms for rapidly controlling protein activity, stability, and interactions in response to cellular signals [7, 8]. Among various PTMs, protein methylation, catalyzed by protein methyltransferases, has emerged as a prevalent and dynamic regulatory mechanism implicated in numerous physiological and pathological processes [9, 10]. While initially characterized for histones in epigenetic regulation, recent advances have revealed that methylation extensively targets non-histone proteins, thereby influencing various critical cellular processes beyond chromatin regulation, including signal transduction, metabolic adaptation, and stress responses [11-13].

The methyltransferase G9a, originally identified as a histone methyltransferase, has now been recognized to demonstrate substantial non-histone methylation activity, significantly expanding its potential biological impacts [14]. It exhibits diverse biological functions across different organs, with roles in liver protection, metabolic regulation, and tissue homeostasis [15-17]. In the kidney, accumulating evidence indicates that G9a promotes renal fibrosis and actively contributes to the pathogenesis of ischemia-reperfusion injury [18, 19]. Our previous work revealed that G9a aggravates RIRI through transcriptional repression of Sirt1 [20]. However, despite these advances, whether G9a regulates diabetic RIRI through methylation represents an important gap.

The FOX transcription factors, particularly FoxO3a, are crucial regulators of cellular homeostasis that coordinate various stress response programs [21-23]. FoxO3a undergoes sophisticated post-translational regulation, including phosphorylation, acetylation, and ubiquitination, that collectively modulates its transcriptional activity, subcellular localization, and protein stability in response to diverse stimuli [24, 25]. In renal injury models, FoxO3a dysfunction has been shown to exacerbate cellular damage through multiple mechanisms, including enhanced inflammatory responses and disruption of redox balance [26, 27]. Despite its established importance in cellular stress responses, the regulatory interplay between methylation and other PTMs in controlling FoxO3a function, particularly in the context of diabetic RIRI, remains largely unexplored and represents a significant knowledge gap.

In this study, we identified G9a as a positive regulator of oxidative stress and pyroptosis in diabetic RIRI. Mechanistically, G9a catalyzed the methylation of the lysine residue of FoxO3a in K262 site in a non-histone form, which leading to the change of subcellular location of FoxO3a. In the cytoplasm, TRIM21 recognizes the methylated FoxO3a and promotes its K48-linked ubiquitination at K176 site. Ubiquitination leads to the degradation of FoxO3a through the proteasome pathway, enhances oxidative stress and pyroptosis, and increases renal injury. Furthermore, BIX‐01294, as a specific G9a inhibitor, exhibits obvious renoprotection effects against IRI in DM, indicating that targeting G9a is extremely promising in clinical application.

**2 Material and methods**

**2.1 Animal experiments**

C57BL/6 mice (20–25g, 6–8 weeks) mice were obtained from the Experimental Animal Center of Wuhan University School of Medicine (Wuhan, China). The study was conducted following the ethical guidelines approved by the Ethics Committee of Renmin Hospital of Wuhan University. Mice were housed in pathogen-free animal facilities maintained at a temperature of 20-25°C, 56% humidity, and a 12/12-hour light/dark cycle with libitum access to water. After one week of adaptation, mice underwent a 12-hour fasting period with free access to water. Blood glucose levels were measured, and body weights were recorded, with blood glucose levels ranging from 5.5-6.5 mmol/L considered normal. Following random grouping, the normal control (NC) group was fed standard chow, while the DM group was fed a high-sugar high-fat diet. After four weeks of feeding, the DM group received 50 mg/kg streptozotocin (STZ; Sigma-Aldrich) by a single intraperitoneal injection, while the NC group received an equivalent volume of citrate buffer intraperitoneally. Fasting blood glucose levels were measured 72 hours later. Successful model establishment was confirmed when mice in the DM group exhibited consecutive three-day blood glucose levels were more than 16.7 mmol/L, accompanied by weight loss, polyuria, polyphagia, and polydipsia.

Kidney-specific G9a conditional knockout (CKO) mice were generated through mating G9a^Flox/Flox^ mice with Cre Cdh16 transgenic mice (Cyagen, Guangzhou, China). A predetermined criterion was established to ensure the validity of knockdown or overexpression efficiency. Experimental data obtained from mice failing to meet these criterions were excluded from analysis.

**2.2 RIRI model**

The establishment of the RIRI model followed previous protocol[28]. Briefly, mice were fully anesthetized using inhalation anesthesia with 2% isoflurane (administered at a rate of 0.6-0.8 L/min), followed by right nephrectomy. Subsequently, the left kidney and renal artery were exposed. Blood flow to the left kidney was occluded using non-traumatic vascular clamps for 30 minutes to induce ischemia, followed by reperfusion at different time points. For the sham operation group, the right kidney was removed, while the blood flow of the left kidney was not blocked. All mice were randomly assigned to four different treatment groups: NC+Sham group, NC+IRI group, DM+Sham group, and DM+IRI group (*n* = 6 in each group).

**2.3 Ethics statement**

All experiments involving animals were conducted according to the ethical policies and procedures approved by the Research Ethics Committee of Renmin Hospital of Wuhan University (Approval no. WDRM20191006).

**2.4 Cell culture and cell H/R model**

The human renal tubular epithelial cells (HK-2, China Center for Type Culture Collection) were cultured in DMEM (Invitrogen, USA) under normal conditions (5% CO_2_, 37°C). The cell H/R model was established using the previously described method[29]. In brief, all cells were randomly assigned to four groups: NC+Con group, NC+H/R group, High Glucose (HG)+Con group, and HG+H/R group. In the NC+Con group, cells were rinsed with D-hanks buffer and then cultured in low-glucose buffer (5.5 mM glucose) for 24 hours. For the NC+H/R group, cells were rinsed with D-hanks buffer and then cultured in low-glucose buffer (5.5 mM glucose) under hypoxic conditions (5% CO_2_, 1% O_2_) for 12 hours, followed by replacement with normal culture medium and incubation under normal conditions (5% CO_2_, 20% O_2_) for 24 hours. In the HG+Con group, cells were rinsed with D-hanks buffer and then cultured in high-glucose buffer (30 mM glucose) for 24 hours. For the HG+H/R group, cells were rinsed with D-hanks buffer and then cultured in high-glucose buffer (30 mM glucose) under hypoxic conditions (5% CO_2_, 1% O_2_) for 12 hours, followed by replacement with normal culture medium and incubation under normal conditions (5% CO_2_, 20% O_2_) for 24 hours.

**2.5 Western blotting (WB) assay**

Total protein from kidney tissues or HK-2 cells were extracted using RIPA buffer (Beyotime, Jiangsu, China). Subsequently, the protein samples were denatured, electrophoresed, and then transferred onto PVDF membranes. Next, the membranes were incubated with corresponding primary antibodies. The primary antibodies used were as follows: FoxO3a (ab47285, Abcam), KIM1 (ab223858, ab213477, Abcam), NGAL (ab216462, Abcam), NLRP3 (ab263899, Abcam), ASC (ab309497, Abcam), Caspase-1 (ab179515, Abcam), IL-1β (ab283818, Abcam), and GAPDH (ab181602, Abcam). These primary antibodies were used in a dilution of 1:200-1:1000. After overnight incubation with the primary antibodies, appropriate secondary antibodies were used, followed by protein band detection using chemiluminescence reagent (Millipore, Billerica, MA, USA).

**2.6 Histological staining**

The kidney tissue was first fixed and embedded for preparation of 4μm thick sections. These sections underwent gradual deparaffinization, hydration, and subsequent stained with hematoxylin and eosin (H&E). The assessment of pathological morphology was evaluated by two expert pathologists who were unaware of the treatment. Histopathological evaluation of tissue damage induced by IRI was performed using the grading scale outlined by Jablonski et al., which ranges from 0 to 4[30].

**2.7 Serum assay**

Measurement was conducted using commercial assay kits and 100 μL of supernatant. All assay kits were utilized in accordance with the manufacturer's instructions (Nanjing Jiancheng Co., China). Serum levels of BUN and Cr were calculated using spectrophotometry method.

**2.8 Superoxide dismutase (SOD) and malondialdehyde (MDA) measurement**

Commercial kits were used in accordance with the manufacturer's instructions (Nanjing Jiancheng Co., China) to measure SOD activity (xanthine oxidase method, catalog#: A001-3) and MDA concentration (thiobarbituric acid method, catalog#: A003-1).

**2.9 siRNA transfection**

For siRNA transfection, HK-2 cells were treated with Lipofectamine 3000 reagent (Invitrogen, Carlsbad, CA, USA) with G9a-specific or FoxO3a-specific siRNA or non-specific siRNA as a negative control. All siRNAs were used at a concentration of 100 nM. After transfection for 48 hours, cells were cultured in DMEM/F12 containing 0.2% FBS for an additional 48 hours. WB assay was conducted to validate the efficiency of siRNA transfection.

**2.10 Adenovirus Transfection**

For overexpression of FoxO3a, HK-2 cells were transfected with adenovirus carrying FoxO3a (Genepharma, Shanghai, China) in DMEM for 12 hours. Subsequently, the medium was replaced with DMEM/F12 containing 10% serum, and cells were cultured. All experiments were conducted in 72 hours after transfection.

**2.11 Adeno-associated virus (AAV9) injection**

The AAV9 delivery system was used to manipulate the expression of FOxO3a gene. Mice were intravenously injected via the tail vein with plasma containing AAV9-mediated overexpression of FoxO3a 2 weeks before IRI. The control group received AAV9 injections simultaneously. Two weeks prior to IRI, AAV9 carrying FoxO3a-targeting shRNA was intravenously injected via the tail vein to silence FoxO3a in CKO mice, with the negative control group receiving AAV9 carrying non-targeting shRNA injections simultaneously. WB assay was performed to validate the efficiency of FoxO3a overexpression or silencing.

**2.12 Immunoprecipitation**

After treatment with lysis buffer, HK-2 cells were scraped and lysed. Subsequently, the lysed cells were centrifuged at 14,000 rpm for 10 minutes at 4°C to obtain total proteins. The proteins were then incubated with corresponding antibodies, introduced to A/G agarose beads, and incubated at 4°C for 3 hours. Following this, the proteins form the beads were eluted and subjected to WB analysis using specified antibodies.

**2.13 Co-immunoprecipitation (Co-IP)**

The cells were collected in RIPA buffer and 2 mg of cell protein was pre-cleared following the above-mentioned method. The cell lysate was incubated with anti-G9a antibody (ab185050, abcam) and anti-FoxO3a antibody (ab47285, abcam), or control IgG, at 4°C for 10 hours. After the precipitate washed, WB assay was performed.

**2.14 Ubiquitination assay**

The expression plasmids of G9a, Myc-ubiquitin, HA-FoxO3a, Myc-ubiquitin (K6O, K11O, K27O, K33O, K48O, K63O) and Flag-G9a were explored for Co-IP detection by transfecting HK-2 cells. The lysine residues related to G9a-mediated ubiquitination of FoxO3a were investigated. The expression plasmids of Myc-ubiquitin (K48R), HA-FoxO3a (WT, K230R, K262R, K290R, K176R, K259R and K360R), Flag-G9a were used for transfection into HK-2 cells for Co-IP assay.

**2.15 RNA sequencing**

Three pairs of kidney tissues in DM group and diabetic RIRI group were used for RNA sequencing. The samples were processed and sequenced by Igenebook Biotech (Wuhan). The results were processed using the R software (version 4.2.1) with limma package. The differentially expressed RNAs were defined as |log_2_FC|>1.5 and P<0.05

**2.16 Bioinformatical analysis**

KEGG analysis and GSEA analysis were performed using R software (version 4.2.1) with ggplot2, stats, car package.

**2.17 Statistic analysis**

Statistical analysis was conducted using GraphPad Prism software (version 9.0, GraphPad Software Inc., USA). Statistical comparisons between multiple groups were conducted using one-way analysis of variance (ANOVA), followed by the Student-Newman-Keuls post hoc test. *P*-value < 0.05 was considered statistically significant.

**3 Results**

**3.1** **DM exacerbated RIRI and up-regulated G9a expression**

First, we established animal model of RIRI under normal and diabetic conditions to confirm the predisposing effect of DM on RIRI. As shown in Figure 1A-D, after RIRI, the renal tubules of mice exhibited dilation, epithelial cell detachment, tubular structural disorder, and disappearance, accompanied by a significant increase in kidney injury score, as well as serum BUN and Cr levels. When establishing RIRI model under DM condition, the above pathological injury manifestation and renal functional impairment were further aggravated, indicating that mice with DM were more susceptible to RIRI. WB results exhibited an increased expression of renal injury markers KIM1 and NAGL proteins after RIRI, which was further significantly increased under DM condition (Figure 1E). In vitro cell H/R injury experiments showed a similar increasing trend in the expression of KIM1 and NAGL proteins in HK2 cell lines cultured in HG (Figure 1F). The increased positive rate of TUNNEL staining confirmed that cell death was induced in cell H/R model under HG condition, accompanied by content increase in MDA and activity decrease in SOD (Figure 1G). Non-histone protein methylation is not well known in diabetic RIRI, thus we performed RNA sequencing to observe the overall expression of methyltransferase. The result of RNA sequencing showed that there are significant changes in the expression levels of many protein methyltransferases (Figure 1H). And KEGG pathway analysis and GSEA enrichment analysis showed that differentially expressed genes were mainly enriched in pathways related to inflammation, cell death, focal adhesion etc. (Figure 1I and J). Based on previous research[20], we chose G9a to testify its expression in diabetic RIRI. WB assay showed that the up-regulation of G9a was enhanced both in the DM mice and HK-2 under HG, which might be mediated by the transcription factor Klf4, and directly binds to and transactivates the G9a promoter (Figure 1K and L; Figure S5).


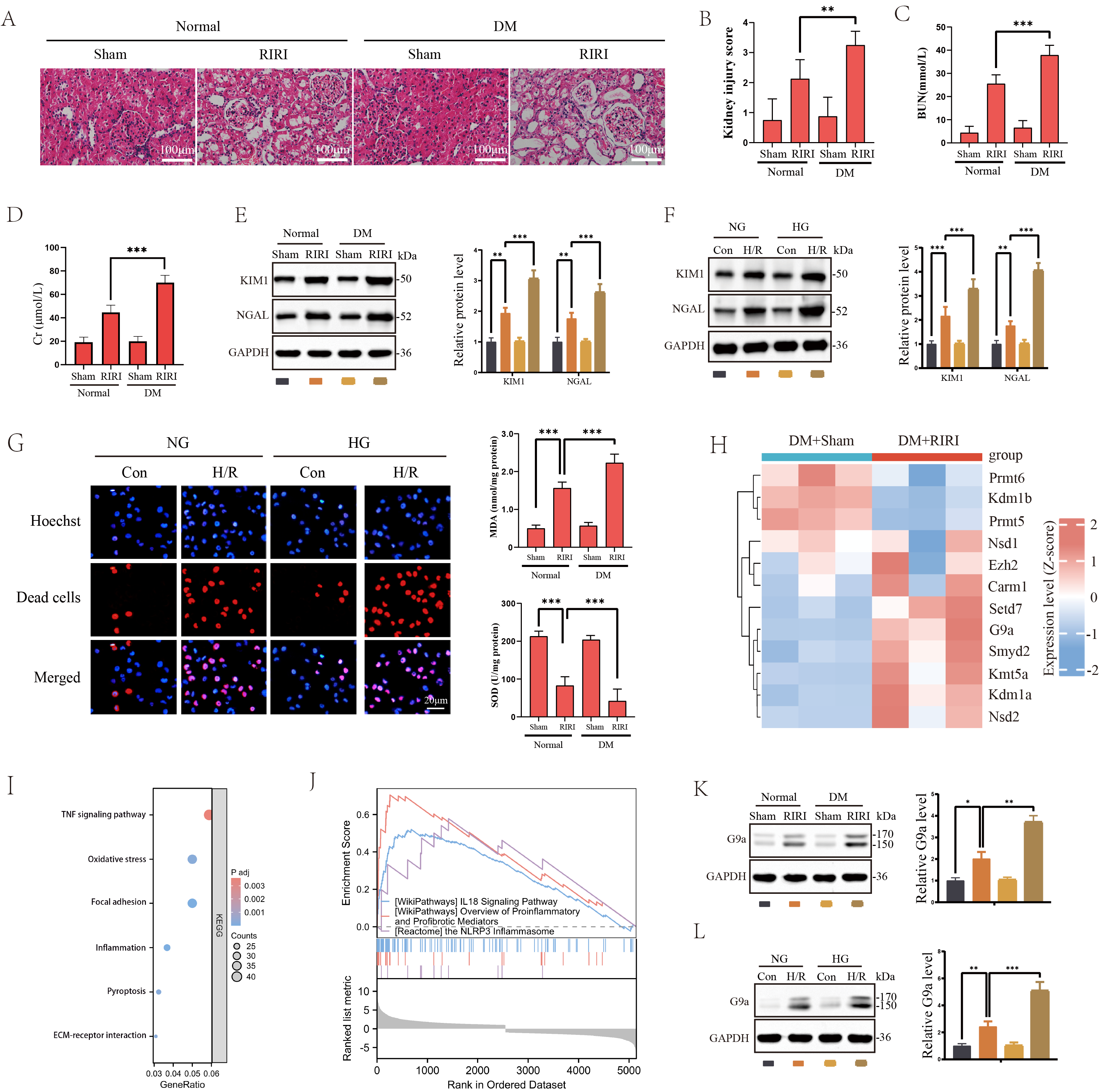


**Figure 1.** DM exacerbated RIRI and up-regulated G9a expression. (A) Representative H&E-stained images of renal tissue slices from the indicated groups (*n* = 6 mice for each group, relative to Sham group). Scale bars, 100μm. (B) Kidney injury score of Sham and RIRI mice under normal or DM condition (*n* = 6 mice for each group). (C) BUN levels of Sham and RIRI mice under normal or DM condition (*n* = 6 mice for each group). (D) Cr levels of Sham and RIRI mice under normal or DM condition (*n* = 6 mice for each group). (E) The levels of renal injury markers KIM1 and NAGL proteins in Sham and RIRI mice under normal or DM condition (*n* = 6 mice for each group). (F) The levels of renal injury markers KIM1 and NAGL proteins in Con and H/R model under normal or HG condition. (G) MDA and SOD production of Sham and RIRI mice under normal or DM condition and representative TUNEL images of dead cells in Con and H/R model under normal or HG condition. Scale bars, 20μm. (H) Heatmap of expression levels of indicated PTMs. (I) KEGG bubble chart of differentially expressed genes. (J) GSEA enrichment analysis of differentially expressed genes. (K) The levels of G9a protein in Sham and RIRI mice under normal or DM condition (n = 6 mice for each group). (L) The levels of G9a protein in Con and H/R model under normal or HG condition. All data are presented as means ± standard deviation (SD). Statistical significance was denoted as **P* < 0.05, ***P*＜0.01 and ****P* < 0.001.

**3.2 Inhibiting G9a alleviated diabetic RIRI and oxidative stress**

RNA sequencing found that the expression levels of some methyltransferase genes with reported non-histone methyltransferase activity showed significant alteration in diabetic RIRI (Figure 1H). Among them, G9a aroused our interest. In our previous study, we have demonstrated that G9a promoted RIRI by repressing Sirt1. However, its function in DM-related RIRI was uncertain. Then we examined the expression levels of G9a in *vivo* and vitro model. G9a levels were increased in both IRI models and further-increased under high-glucose condition (Figure 1K, L). To elucidate the regulatory role of G9a during DM-related RIRI, we generated conditional knockout (CKO) mice for G9a in renal tubular epithelial cells, validated the mouse genotype through PCR and confirmed the knockout effect through WB assay (Figure 2A, B, C and D). The CKO mice exhibited reduced pathological damage compared to G9a^Flox/Flox^ mice after IRI treatment, accompanied by reduction in kidney injury score (Figure 2E, F), serum BUN and Cr levels (Figure 2G, H), as well as expression levels of KIM1 and NAGL protein, indicating that G9a knockout significantly alleviated RIRI (Figure 2I). It showed that silencing G9a or inhibiting G9a activity with BIX resulted in decreased expression of KIM1 and NAGL protein and cell death rate, accompanied by content decrease in MDA and activity increase in SOD, indicating alleviation of oxidative stress and renal tubular epithelial cell injury (Figure 2J-M).


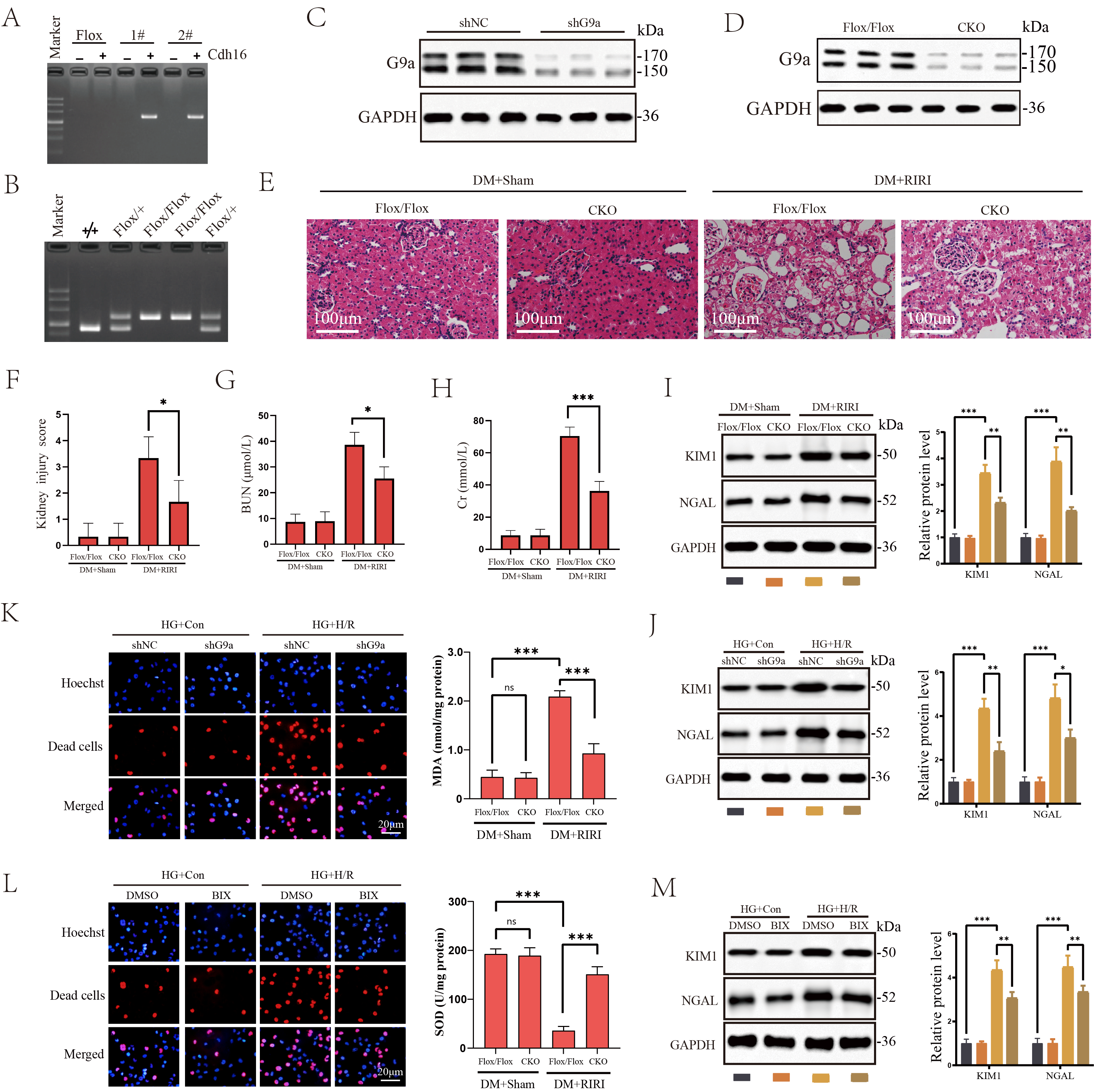


**Figure 2.** Inhibiting G9a alleviated diabetic RIRI. (A, B) The PCR of G9a^Flox/Flox^ and Cdh16-Cre mice, gel electrophoresis imaging showed that the length of Cre gene and Flox gene amplification product. (C) Representative WB images of G9a expression in HK-2 cells treated with Control shRNA or shG9a. (D) WB results validated the genotype of Flox/Flox and CKO mice. (E) Representative H&E staining images of renal tissue slices from the CKO groups (*n* = 6 mice for each group, relative to Flox/Flox group). Scale bars, 100μm (F) Kidney injury score of Flox/Flox and CKO mice under DM condition from the indicated groups (*n* = 6 mice for each group, relative to Sham group). (G) BUN levels of Flox/Flox and CKO mice under DM condition (*n* = 6 mice for each group, relative to Sham group). (H) Cr levels of Flox/Flox and CKO mice under DM condition from the indicated groups (*n* = 6 mice for each group, relative to Sham group). (I) The levels of renal injury markers KIM1 and NAGL proteins in Flox/Flox and CKO mice under DM condition from the indicated groups (*n* = 6 mice for each group, relative to Flox/Flox group). (J) The levels of renal injury markers KIM1 and NAGL protein in shRNA and shG9a groups in Con and H/R model under HG condition. (K) MDA production of Flox/Flox and CKO mice under DM condition and representative TUNEL images of dead cells in shRNA and shG9a groups in Con and H/R model under HG condition. Scale bars, 20μm. (L) SOD production of Flox/Flox and CKO mice under DM condition and representative TUNEL images of dead cells in groups with DMSO and BIX in Con and H/R model under HG condition. Scale bars, 20μm. (M) The levels of renal injury markers KIM1 and NAGL protein in groups with DMSO and BIX in Con and H/R model under HG condition. All data are presented as means ± SD. Statistical significance was denoted as **P* < 0.05, **P＜0.01 and ****P* < 0.001.

**3.3** **G9a promoted cell pyroptosis and targeted FoxO3a in diabetic RIRI**

To further elucidate the potential mechanism of G9a regulation in diabetic RIRI, we conducted mass spectrometry (MS) experiment and Co-IP assay to identify the proteins interacting with G9a and performed KEGG enrichment analysis on the interacting protein. Through MS experiments, we identified FoxO3a as a downstream target of G9a action, and KEGG analysis showed that the pyroptosis pathway was significantly enriched among all interacting proteins (Figure 3A and B). We assessed changes in cell viability using the Necrostatin-1, ABT-199 and VX-765, and found that KIM1 and NAGL protein significantly decreased after treatment with the pyroptosis inhibitor, while the cell viability was significantly higher (Figure S6). Meanwhile, both in *vivo* and in vitro models, observations revealed that increased expression of pyroptosis-related markers NLRP3, ASC, Cle-caspase-1, and IL-1β, and their expression was further promoted under DM condition (Figure 3C and S1A). Collectively, we found that susceptibility to RIRI was increased under DM condition, and elevated G9a expression promoted cell pyroptosis, suggested a potential link between G9a and cell pyroptosis in DM-related RIRI. Furthermore, during the process of RIRI, the CKO mice exhibited significantly downregulated expression of NLRP3, ASC, Cle-caspase-1, and IL-1β proteins compared to G9a^Flox/Flox^ mice (Figure 3D). Silencing G9a or inhibiting G9a activity with BIX led to significant downregulation of pyroptosis-related proteins in vitro (Figure S1B and 3E). In combination, these results demonstrated that G9a participated in the regulation of cell pyroptosis during DM-related RIRI both in *vivo* and in vitro. Subsequent Co-IP experiments with overexpressed G9a and FoxO3a in 293T cells and GST pull-down assays confirmed the direct interaction between G9a and FoxO3a (Figure F-H). Furthermore, we determined the interaction domains between G9a and FoxO3a by expressing truncated mutants of G9a and FoxO3a. Co-IP results showed that the G9a protein 680-896 amino acid (aa) interacted with the FoxO3a protein 150-300aa (Figure 3I and J).


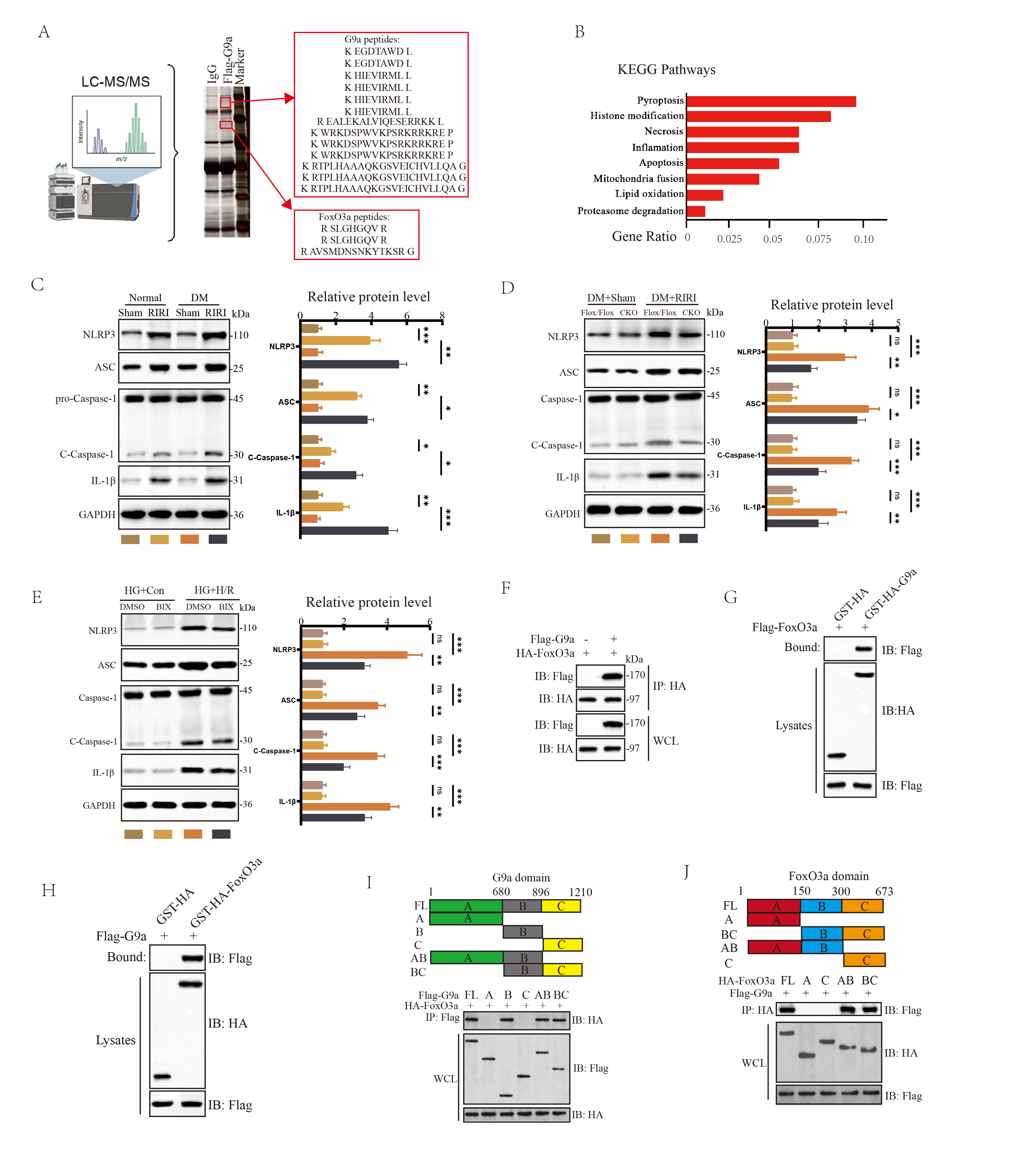


**Figure 3.** G9a promoted cell pyroptosis and targeted FoxO3a in diabetic RIRI. (A) MS analysis indicating potential G9a-interacting proteins. (B) Key biological pathways associated with function of G9a-interacting proteins were identified through KEGG enrichment analysis. (C) The levels of pyroptosis-related proteins in Sham and IRI mice under normal or DM condition (n = 6 mice for each group). (D) The levels of pyroptosis-related proteins in Flox/Flox and CKO mice under DM condition from the indicated groups (*n* = 6 mice for each group, relative to Sham group) (E). (F) The levels of pyroptosis-related proteins in groups with DMSO and BIX in Con and H/R model under HG condition. Co-IP assays conducted to evaluate the interaction between G9a and FoxO3a. (G, H) GST pull-down assays confirmed the direct interaction between G9a and FoxO3a. (I) (J) Co-IP analysis investigating the interaction domains between G9a and FoxO3a. **P* < 0.05, **P＜0.01 and ****P* < 0.001

**3.4** **Degradation of FoxO3a was promoted via methylation by G9a**

Having established the direct interaction between G9a and FoxO3a, we investigated the regulatory role of G9a in FoxO3a expression in DM-related RIRI. First, in animal experiments, we observed a significant decrease in FoxO3a protein expression in the DM+IRI group compared to the DM+Sham group following RIRI (Figure S2A). Similar results were observed in in vitro cell experiments, where FoxO3a levels decreased more significantly in the HG condition of the cell H/R model (Figure 4A). In DM-related RIRI, CKO mice showed upregulation of FoxO3a protein expression compared to G9aFlox/Flox mice (Figure S2B). Furthermore, in the H/R model, we found that silencing G9a or inhibiting G9a activity with BIX resulted in significant upregulation of FoxO3a protein expression (Figure 4B and C). Through in vitro overexpression system, we found that G9a could directly methylate FoxO3a (Figure 4E). In the in *vivo* model and cell H/R model, DM-related RIRI significantly promoted FoxO3a methylation, aligning with findings from in vitro overexpression experiments (Figure S2C and 4D). It is worth noting that the acetylation status of FoxO3a does not regulate the methylation process (Figure S9). In the 150-300aa region, we predict through the database that there are three methylated lysine residues, namely K230, K262 and K290. Therefore, we attempted to determine the specific methylation site of FoxO3a by G9a through site-directed mutagenesis. The results indicated that K262 was the site where G9a methylated FoxO3a (Figure 4F). Since the interaction between G9a and FoxO3a has been established, we further investigated how G9a regulated the stability of FoxO3a. We treated HK2 cells with autophagy-lysosome inhibitor CQ and proteasome inhibitor MG132 and detected FoxO3a protein expression by WB. The findings indicated that CQ did not maintain the stability of FoxO3a protein, while MG132 significantly stabilized FoxO3a protein (Figure 4J). Moreover, we found that silencing G9a promoted the stability of FoxO3a protein. Time-course analysis further demonstrated that G9a reduced the half-life of endogenous FoxO3a (Figure 4K). These results indicated that G9a regulated FoxO3a protein expression through the proteasome-ubiquitin system. In addition, one of the major functions of G9a is as a transcription regulator. We also examined the effect of G9a on FoxO3a mRNA in HK-2 cells, and the results showed that neither shG9a nor BIX treatment affected the transcription of FoxO3a (Figure G-I). Taken together, these findings demonstrated that G9a interacted with FoxO3a in DM-related RIRI through direct methylation of FoxO3a at the K262 site, thereby promoting the protease system degradation of FoxO3a.


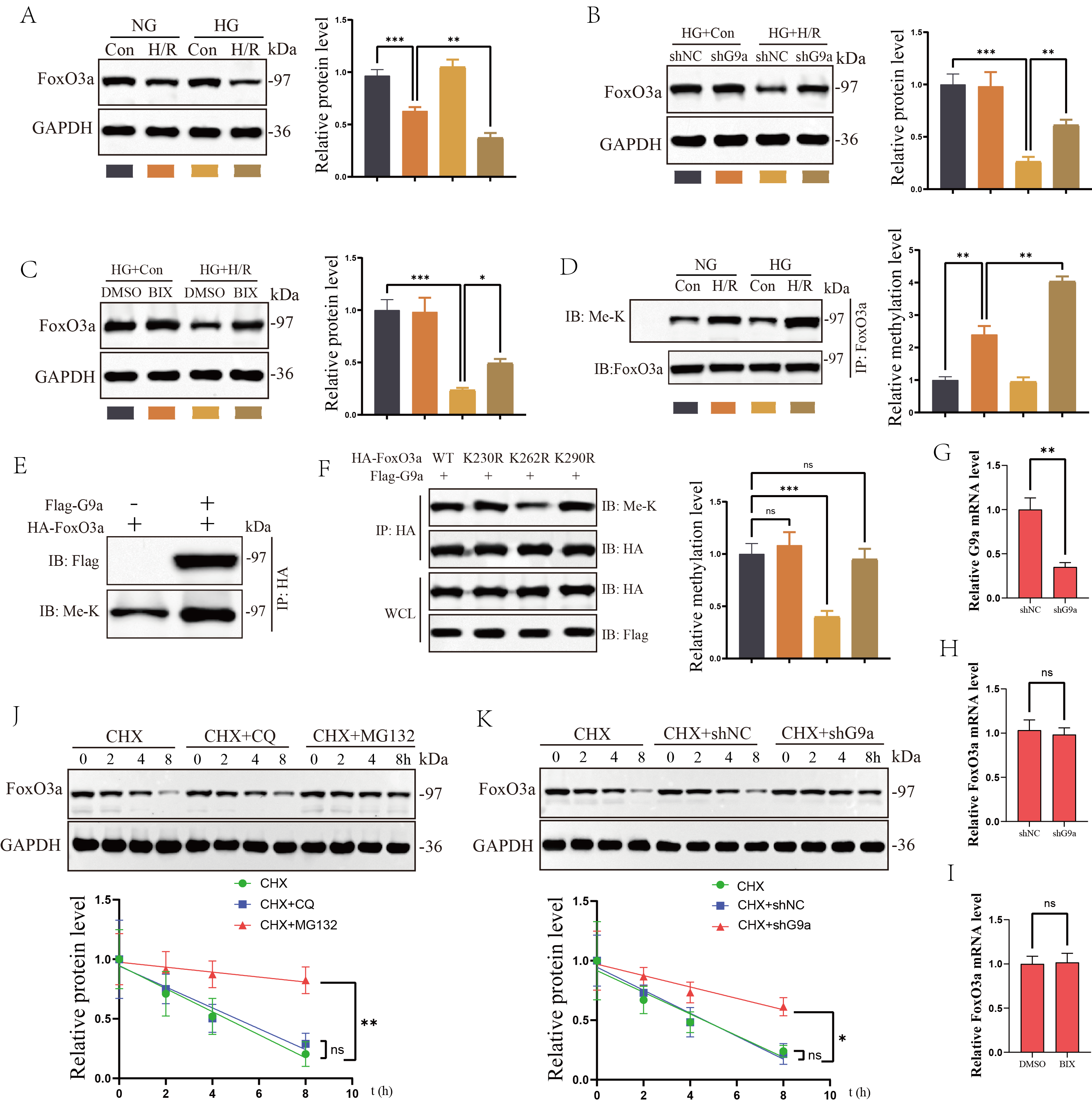


**Figure 4.** Degradation of FoxO3a was promoted via methylation by G9a. (A) The levels of FoxO3a protein in Con and H/R model under normal or HG condition. (B) The levels of FoxO3a protein in shRNA and shG9a groups in Con and H/R model under HG condition. (C) The levels of FoxO3a protein in groups with DMSO and BIX in Con and H/R model under HG condition. (D) Representative WB images showed that H/R promoted the methylation of FoxO3a in cell H/R model. (E) Representative WB images showed that G9a methylated FoxO3a in *vitro* overexpression experiments. (F) Representative WB images of methylation level of indicated HA-FoxO3a. (G) Relative mRNA levels of G9a in shNC and shG9a group. (H) Relative mRNA levels of FoxO3a in shNC and shG9a group. (I) Relative mRNA levels of FoxO3a in DMSO and BIX group. (J) WB analysis of FoxO3a protein stability in HK2 cells treated with CQ or MG132. (K) WB analysis of FoxO3a protein stability with shRNA or shG9a in the in HK-2 cells. All data are presented as means ± SD. Statistical significance was denoted as **P* < 0.05, **P＜0.01 and ***P＜0.001.

**3.5** **G9a promoted K48-linked ubiquitination of FoxO3a at the K176 site and proteasomal degradation**

Further studies revealed that ubiquitination of FoxO3a increased in the diabetic RIRI in *vivo* and vitro (Figure 5A and B), while deletion or silencing of G9a reduced the ubiquitination of FoxO3a (Figure 5C and D). As different types of polyubiquitin linkages mediate different biological functions, we expressed HA-tagged FoxO3a and various ubiquitin (K6O, K11O, K27O, K33O, K48O, and K63O) in HK-2 cells, each containing only one specified lysine residue available for polyubiquitin chain formation. Ubiquitination results showed that G9a enhanced FoxO3a ubiquitination degradation through the formation of K48-linked chain (Figure 5E). When the K48 ubiquitination site was mutated, G9a-mediated FoxO3a ubiquitination was abolished (Figure 5F). Additionally, after mutating the methylated modification site K262 of FoxO3a and transfecting it into 293T cells, we found that FoxO3a ubiquitination was significantly inhibited after methylation inactivation (Figure 5G). Subsequently, through bioinformatics analysis, we identified three potential ubiquitination sites on the FoxO3a protein and then detected the ubiquitination sites of FoxO3a after mutating each of the three sites. The results showed that K176 was the ubiquitination modification site of FoxO3a (Figure 5H). These findings strongly proved that G9a promoted FoxO3a degradation through the proteasome-ubiquitin system, and K48 was an important site for FoxO3a ubiquitination and stability. We observed the subcellular localization of G9a and FoxO3a through immunofluoresence double staining. The result showed that FoxO3a showed a tendency for translocation under the activation of WT-G9a in diabetic RIRI, which was eliminated by K262R-G9a (Figure 5I). Collectively, these findings provided compelling evidence that G9a regulated FoxO3a protein methylation during diabetic RIRI, which promoting the ubiquitination and translocation of FoxO3a.


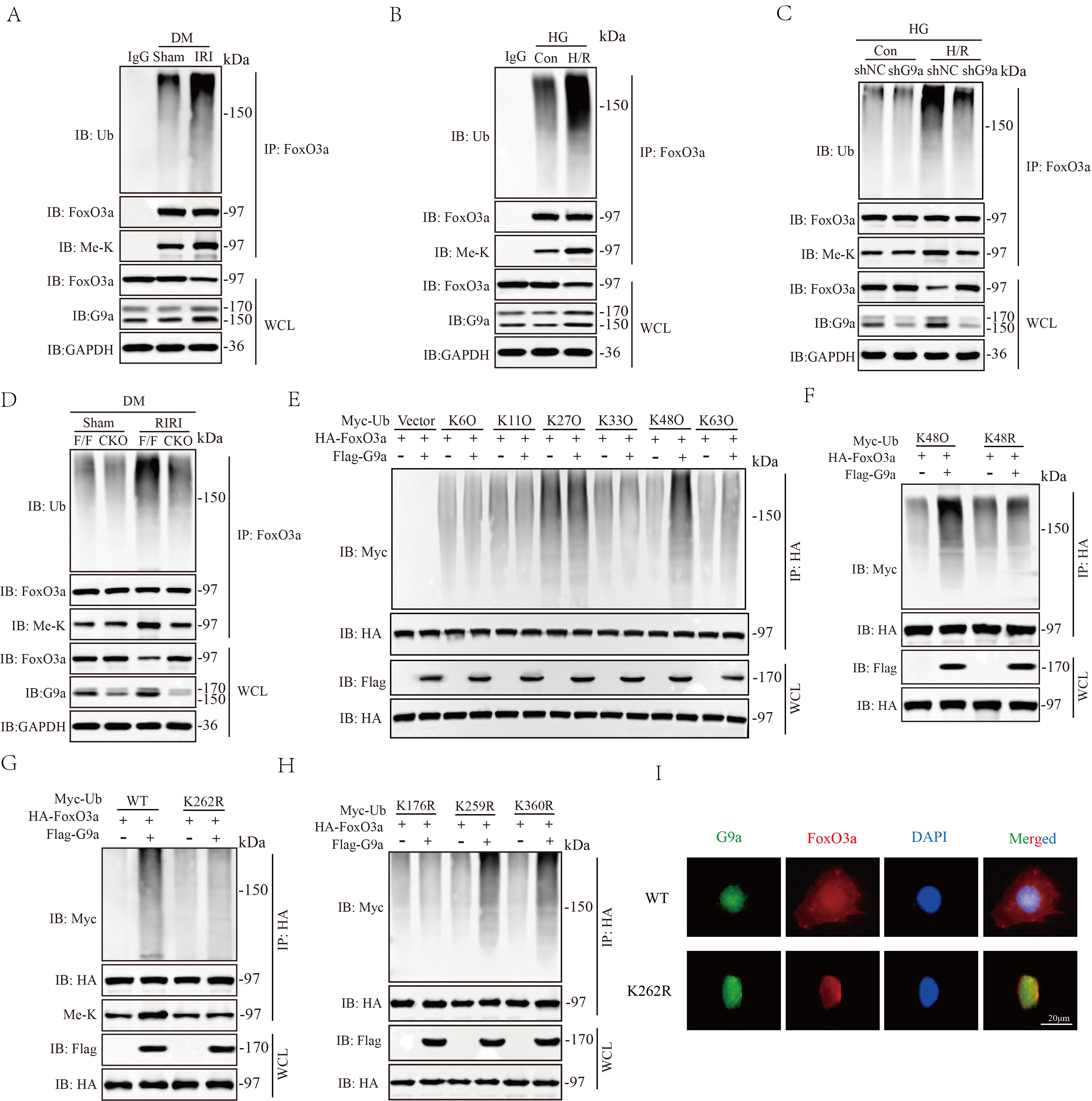


**Figure 5.** G9a promoted K48-linked ubiquitination of FoxO3a at the K176 site and proteasomal degradation. (A) Co-IP analysis of FoxO3a ubiquitination in IRI and control groups. (B) Co-IP analysis of FoxO3a ubiquitination in Con and H/R groups. (C) Co-IP analysis of FoxO3a ubiquitination in IRI mice of G9a knockdown and Flox/Flox groups. (D) Co-IP analysis of FoxO3a ubiquitination in cell H/R model of using shRNA and shG9a groups. (E) Ubiquitination screening of FoxO3a by G9a using different types of ubiquitin (K6O, K11O, K27O, K33O, K48O, and K63O). (F) Ubiquitination of FoxO3a in HK-2 cells transfected with G9a and indicated ubiquitin mutant K48R plasmids. (G) Ubiquitination of FoxO3a in 293T cells after mutating the methylated modification site K262. (H) Ubiquitination site screening of FoxO3a after mutating different types of ubiquitination modification site (K176R, K259R and K360R). (I) Immunofluorescence analysis demonstrating the localization of G9a and FoxO3a under cell H/R model with WT or K262R FoxO3a, Scale bars, 10μm.

**3.6** **FoxO3a alleviated diabetic RIRI and involved in driving diabetic RIRI by G9a**

Next, we utilized AAV9 to overexpress FoxO3a in *vivo* to investigate the pivotal role of FoxO3a in diabetic RIRI. (Figure 6A). The results revealed that overexpression of FoxO3a in DM+IRI group led to a significant reduction in serum levels of BUN, Cr and MDA, and an increase of SOD content, alleviated histopathological damage to renal tubular epithelial cells, and reduced the expression of renal injury markers KIM1 and NAGL, thereby significantly mitigating tissue damage and renal dysfunction in RIRI (Figure 6B-E). WB assays indicated that overexpression of FoxO3a during RIRI significantly inhibited the expression of NLRP3, ASC, Cle-caspase-1, and IL-1β proteins (Figure 6F). In line with the in *vivo* observations, the overexpression of FoxO3a in the in vitro cell H/R model demonstrated a similar trend, showing a significant downregulation of pyroptosis-related protein expression in an HG condition during H/R injury (Figure S3A, B). Figure S3C illustrated the increased number of deceased cells in the cell H/R model under HG conditions, confirming the induction of pyroptosis. In conclusion, these findings provided compelling evidence that FoxO3a could alleviate diabetic RIRI and induced oxidative stress.

To elucidate whether G9a regulated DM-related RIRI through FoxO3a, we silenced FoxO3a in G9a knockout mice using AAV9. Our results showed that silencing FoxO3a resulted in a notable elevation in serum Cr and BUN, tubular dilation, and epithelial cell shedding, further exacerbating renal injury (Figure 6G-K). Besides, it was observed that silencing FoxO3a significantly increased the expression of renal injury markers KIM1 and NAGL, alongside the pyroptotic markers NLRP3, ASC, Cle-caspase-1, and IL-1β (Figure 6L). Meanwhile, the silencing of FoxO3a resulted in an increase in the levels of Cr, BUN and MDA, and a decrease of SOD content (Figure 6J-K). In vitro experiments using stable G9a knockdown cell lines further confirmed these findings, showing that silencing FoxO3a in H/R model significantly increased the expression of renal injury and pyroptosis-related proteins, consistent with the in *vivo* results (Figure S3E-G). The increased number of dead cells confirmed that pyroptosis was induced by silencing FoxO3a in G9a knockdown cell H/R model under HG condition (Figure S3D). Furthermore, we found that G9a-knockdown enhanced protective effect conferred by wild-type FoxO3a was effectively abolished by a degradation-resistant FoxO3a mutant (K176R). Conversely, in a G9a-overexpression background, the degradation-resistant mutant failed to rescue the injury phenotype, underscoring that a functional G9a-FoxO3a regulatory interaction is indispensable for mediating pyroptosis and renal injury (Figure S8). In summary, these results provided evidence for the potential involvement of the G9a-FoxO3a axis in promoting cellular pyroptosis and oxidative stress during the progression of diabetic RIRI.


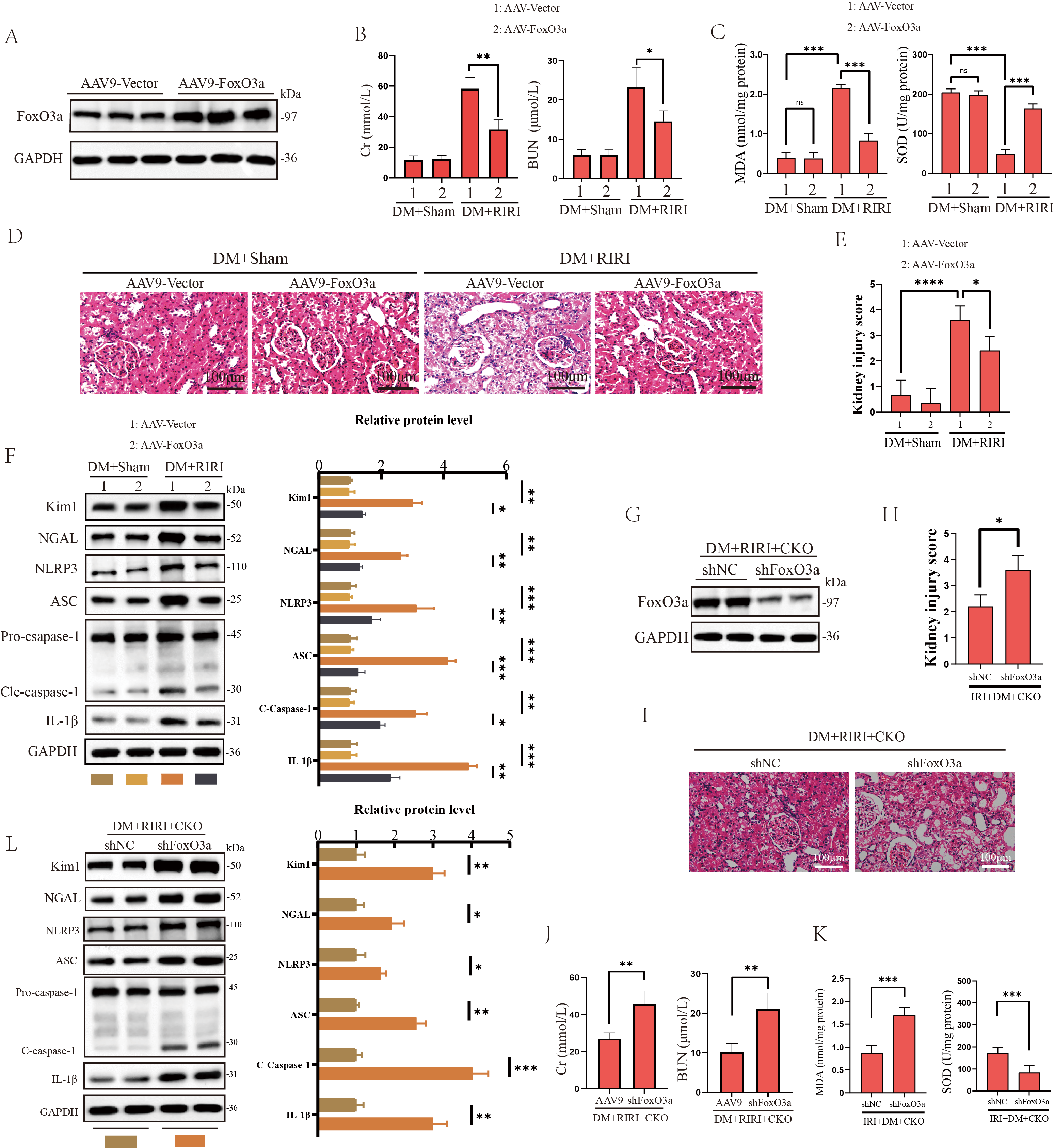


**Figure 6.** FoxO3a alleviated diabetic RIRI and involved in driving diabetic RIRI by G9a. (A) WB analysis of FoxO3a protein expression by AAV9 in the *vivo*. (B) Cr and BUN levels of FoxO3a overexpression in Sham and IRI mice under DM condition (*n* = 6 mice for each group). (C) MDA and SOD production of FoxO3a overexpression in Sham and IRI mice under DM condition. (D) Representative H&E staining images of renal tissue slices from mice in the indicated groups (*n* = 6 mice for each group). Scale bars, 100μm. (E) Statistics analysis of kidney injury score in indicated groups. (F) The levels of KIM1 and NAGL as well as pyroptosis-related proteins in the indicated groups (*n* = 6 mice for each group). (G) WB analysis of FoxO3a protein expression in the G9a-CKO mice treated by AAV9-shFoxO3a during DM-related RIRI. (n = 6 mice for each group). (H) Statistics analysis of kidney injury score in indicated groups. (I) Representative H&E staining images of renal tissue slices from CKO mice in the indicated groups (n = 6 mice for each group). Scale bars, 100μm. (J) Cr and BUN levels of CKO mice in the indicated groups (n = 6 mice for each group). (K) MDA and SOD production of FoxO3a silencing in Sham and IRI mice under DM+CKO condition. (L) The levels of KIM1 and NAGL proteins as well as pyroptosis-related proteins in the indicated groups (n = 6 mice for each group). All data are presented as means ± SD. Statistical significance was denoted as **P* < 0.05 and ***P* < 0.01 and ***P＜0.001.

**3.7** **G9a facilitated the ubiquitination of FoxO3a mediated by TRIM21**

To further elucidate the mechanism of action of G9a, we conducted an IP screening of multiple TRIM proteins to identify the E3 ubiquitin ligase associated with FoxO3a. In the initial screening, TRIM21, TRIM27, and TRIM231 demonstrated bands in the IP linked to FoxO3a (Figure 7A, B and S4A, B). Consequently, these three TRIM proteins were selected for Co-IP ubiquitination assays. The results indicated that only TRIM21 enhanced the ubiquitination of FoxO3a (Figure 7C) and the ubiquitination site is K176(Figure 7K). The cycloheximide CHX assay demonstrated that the inhibition of TRIM21 effectively extended the half-life of FoxO3a (Figure 7F). To further determine the precise binding region of TRIM21 and FoxO3a, we enforced the expression of full-length and fragmented HA-FoxO3a and Flag-TRIM21 in 293T cells. Through a Co-IP assay, we found that TRIM21 bound to the C-terminal (amino acids 128-475) domain of FoxO3a and was recognized by FoxO3a at N-terminal (amino acids 1-148). To elucidate the relationship between G9a methylation and TRIM21-mediated ubiquitination, we conducted a series of comprehensive experiments. Our findings indicate that ischemic conditions significantly enhance the ubiquitination of FoxO3a, which can be abrogated by conditional knockout of G9a (Figure 7G). Furthermore, cellular assays demonstrated that TRIM21 knockdown inhibited FoxO3a ubiquitination under H/R conditions, while the methylation status of FoxO3a remained unchanged (Figure 7H). Contrarily, the overexpression of TRIM21 enhances the ubiquitination of FoxO3a under H/R conditions. However, the reduction in FoxO3a methylation levels resulting from G9a knockout concurrently impedes TRIM21-mediated ubiquitination of FoxO3a (Figure 7I). Experiments involving the transfection of wild-type FoxO3a, a methylation site mutant K262R, and a ubiquitination site mutant K176R into 293T cells revealed that the methylation inhibition induced by the K262 mutation abolished ubiquitination. In contrast, the K176R mutation inhibited ubiquitination without affecting the methylation status of FoxO3a (Figure 7L). Immunofluorescence analysis revealed that K262R is capable of restricting the nuclear translocation of FoxO3a, whereas K176R does not impede the colocalization of FoxO3a with TRIM21 (Figure 7J). Collectively, these findings suggest that TRIM21 facilitates the ubiquitination of FoxO3a and its activity is modulated by G9a-mediated methylation.


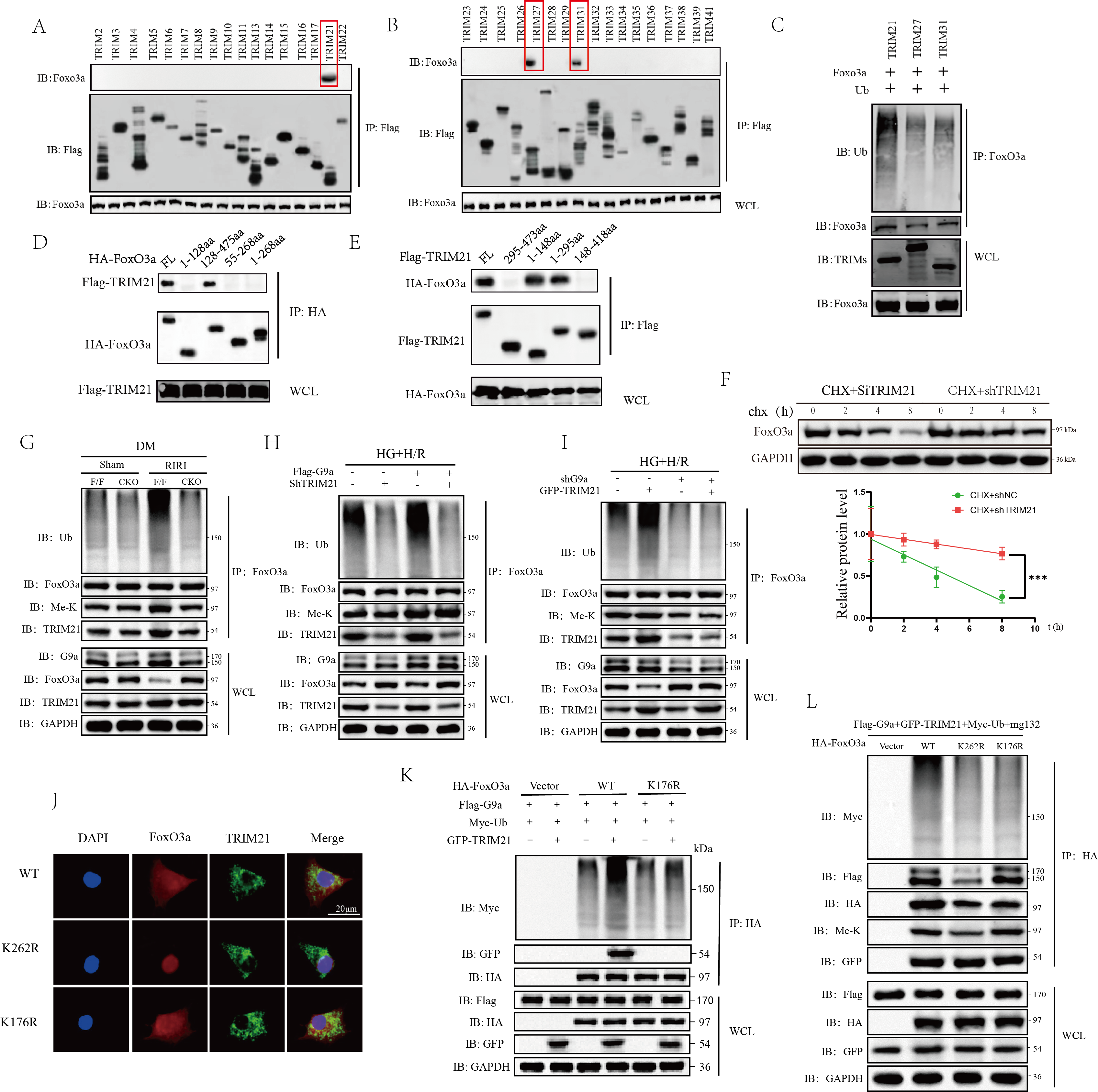


Figure **7.** G9a facilitated the ubiquitination of FoxO3a mediated by TRIM21. (A) (B) WB analysis of IP assay between FoxO3a and TRIM proteins. (C) WB analysis of FoxO3a ubiquitination IP assay. (D)(E) Co-IP analysis investigating the interaction domains between TRIM21 and FoxO3a. (F) WB analysis of FoxO3a protein expression CHX and shTRIM21 treatment in the cell H/R model. (G-I) WB analysis of FoxO3a ubiquitination and methylation IP assay in indicated groups. (J) Immunofluorescence analysis demonstrating the localization of TRIM21 and FoxO3a under cell H/R model with WT, K262R or K176R FoxO3a. Scale bars, 10μm. (K)(L) WB analysis of FoxO3a ubiquitination and methylation IP assay in indicated groups. All data are presented as means ± SD. Statistical significance was denoted as ***P＜0.001.

**4 Discussion**

Previous investigations into protein methylation have predominantly concentrated on histone modifications and have established histone methylation as a key indicator of chromatin state and gene expression status in many diseases[31, 32]. However, the increasing functions of non-histone methylation in cellular pathways and protein activity has recently been revealed and aroused attention. The tumor suppressor protein p53 is well-recognized for its role in inhibiting tumorigenesis; however, it is important to acknowledge that it has been reported to undergo methylation by various PKMTs. Specifically, G9a has been shown to methylate p53 at lysine 373, thereby repressing DNA damage repair and suggesting that G9a may constrain p53 activity[33]. Conversely, SETD7 monomethylates p53 at lysine 372, which positively regulates the stability of the p53 protein[34]. Additionally, SMYD2 and SETD8 target other lysine residues on p53, influencing its activity[35, 36]. Protein methylation also plays a crucial role in mitochondrial function associated with Alcoholic Fatty Liver Disease (AFLD). Alcohol consumption leads to elevated levels of both MAT1α and S-adenosylmethionine (SAM), which in turn enhances protein lysine methylation. This process results in the upregulation of several key mitochondrial proteins that are integral to the tricarboxylic acid (TCA) cycle, β-oxidation of fatty acids, and oxidative phosphorylation pathways, ultimately contributing to AFLD[37]. Furthermore, another recent research has demonstrated that SETD7 can methylate Yes-associated protein (YAP) during myocardial ischemia, promoting its retention in the cytosol and diminishing the transcription of antioxidant genes such as manganese superoxide dismutase (MnSOD) and catalase (CAT). This mechanism leads to myocardial injury and dysfunction of cardiomyocytes[38].

The targeting of non-histone by numerous PKMTs suggests that non-histone methylation is prevalent and plays a significant role in essential pathways, including responses to genotoxic stress, oxidative stress, inflammation. Analogous to histone methylation, the principal role of non-histone methylation is to modulate protein–protein interactions, encompassing protein stability, subcellular localization, and DNA binding[39]. Despite its significant potential, the role and mechanisms of non-histone methylation in diabetic RIRI remain unclear. To gain a comprehensive understanding of the status of non-histone methylation in this context, we conducted RNA sequencing on renal ischemia mouse models with diabetes. The results revealed substantial alterations in the expression of multiple known methyltransferases capable of non-histone methylation. G9a aroused our strong interest among the genes exhibiting altered expression, as our previous research demonstrated that G9a modulates renal ischemia-reperfusion (IR) injury through its interaction with CBX1 to form a transcriptional repressor complex on the Sirt1 promoter, thereby regulating the generation of superoxide (O2·-) and hydroxyl radicals (·OH). Nonetheless, further investigation is required to elucidate its role in non-histone protein methylation and diabetes mellitus-related ischemic injury. To achieve this, we first assessed the role of G9a utilizing animal models of RIRI in DM and cell H/R model in HG. The results indicated that DM as a susceptibility factor exacerbate the upregulation of G9a in IRI and inhibiting G9a could attenuate the damage and oxidative stress induced by IRI both in *vivo* and in vitro.

We initially identified downstream protein interacting with G9a using MS assay and performed KEGG analysis on these downstream proteins, revealing pyroptosis as the most enriched pathway. In recent decades, investigations into the mechanisms underlying RIRI have primarily concentrated on various forms of programmed cell death, encompassing pyroptosis, ferroptosis, and apoptosis. These distinct modes of cell death play pivotal roles in the pathogenesis of AKI[40-42]. Notably, pyroptosis is acknowledged as the predominant inflammatory cell death pathway implicated in this context[43]. Typical pyroptosis is mediated by inflammasomes, which consist of sensors, adapters, and effectors, such as caspase 1, 4, 5 and 11. Among the various inflammasome complexes, the NLRP3 inflammasome is notably recognized as the principal mediator of pyroptosis[44]. Activation of the NLRP3 inflammasome leads to gasdermin D-mediated cell swelling and release of mature pro-inflammatory cytokines, typically IL-1β and IL-18[45]. Consistent with these studies, the results of KEGG analysis and GSAE enrichment analysis showed that pyroptosis, NLRP3 inflammasome and IL-18 signalling pathway is significantly up-regulated in diabetic RIRI, which further confirmed that pyroptosis may play critical roles in diabetic RIRI. In subsequent experiments, G9a was found to promote the levels of NLRP3, ASC, Cle-caspase-1, and IL-1β proteins in the kidneys of IRI-induced AKI. Furthermore, inhibition of G9a using BIX or G9a knockout mice attenuated diabetic RIRI, significantly reducing NLRP3 and IL-1β expression, accompanied by a decrease in MDA level and an increase in SOD level, thus confirming G9a role in promoting oxidative stress and cell pyroptosis mediated by NLRP3.

In the MS assay, significant interaction between FoxO3a and G9a was observed. Further GST pull-down experiments demonstrated a direct interaction between G9a and FoxO3a, and Co-IP was used to determine the binding domain responsible for the G9a-FoxO3a interaction. And our study revealed a significant upregulation of FoxO3a methylation and concomitant suppression of its expression in diabetic RIRI both in *vivo* and vitro. Inhibition of G9a resulted in a marked downregulation of FoxO3a methylation levels and a restoration of its expression. Through analysis of the interacting fragments, we finally identified K262 site as the specific site of G9a-mediated methylation on FoxO3a, among the three potential sites (K230, K262, K290).

Research has shown that FoxO3a is deeply involved in the regulation of pyroptosis. Upon exposure to infection or various stressors, the activity of FoxO3a can be modulated, influencing the activation of NLRP3 inflammasomes, resulting in the intracellular release of inflammatory mediators that subsequently initiate pyroptosis[46]. Furthermore, FoxO3a may influence the induction of pyroptosis through its regulation of the oxidative stress response. Oxidative stress is a significant factor in the initiation of pyroptosis, and FoxO3a is integral to the cellular response to such stress[47]. Consequently, the function of FoxO3a in modulating cell pyroptosis extends beyond gene expression regulation and is potentially intricately linked to the metabolic status and redox equilibrium of cells. Therefore, FoxO3a is highly likely to be the target of G9a for mediating pyroptosis and oxidative stress in diabetic RIRI. Our further findings revealed opposite expression pattern of G9a and FoxO3a in DM-related RIRI.

In *vivo* findings demonstrated that tail vein injection of AAV9-shRNA targeting FoxO3a significantly re-aggravated renal injury alleviated by G9a CKO. In vitro, gene inhibition of FoxO3a with siRNA disrupted the cellular protective effect provided by G9a knockdown against H/R stimulation under HG environment. These results demonstrated the involvement of FoxO3a in G9a mediated renal damage in diabetic RIRI. The interaction between G9a and FoxO3a is one novelty of our study, which was crucial for understanding how these genes dynamically promoted ischemic diseases under DM conditions.

Numerous studies indicate that epigenetic modifications play a role in regulating protein stability. To delve deeper into the molecular mechanism of G9a regulation of FoxO3a, we investigated the degradation pathway of FoxO3a and found that FoxO3a degradation was associated with the proteasomal system. Ubiquitin-proteasome degradation, one of the two primary pathways for protein degradation, plays a pivotal role in regulating various biological processes[48]. In our experiments, we noted a significant elevation in FoxO3a ubiquitination during IRI, while its ubiquitination was significantly reduced when G9a was silenced or knocked out. Ubiquitin consists of seven lysine residues, each of which serves as a potential linkage site for ubiquitin chains. The fate of substrate proteins undergoing ubiquitination largely hinges on the specific lysine residue to which ubiquitin is attached. To elucidate the ubiquitination pattern of FoxO3a, we constructed ubiquitin K6O, K11O, K27O, K33O, K48O, and K63O for co-immunoprecipitation assays. Our results unveiled that G9a promoted K48-linked chain ubiquitination and degradation of FoxO3a. After constructing the K48-mutation ubiquitin plasmid, there was no significant change in the ubiquitination level. We further constructed FoxO3a mutants K176R, K259R, and K360R based on site prediction to verify which sites mediate G9a-induced ubiquitination degradation of FoxO3a. Co-IP detection showed that G9a catalyzed K48-linked polyubiquitination degradation of FoxO3a-K176R, but not FoxO3a-K259R and FoxO3a-K360R, confirming for the first time the important role of G9a in ubiquitination degradation of FoxO3a at the K176 site. In cardiac IRI, Das et al. found elevated levels of G9a protein and histone methylation concurrently with nuclear translocation of FoxO3a in Caveolin knockout mice[27]. Thus, double-immunofluorescence staining was employed to examine the subcellular localization of G9a and FoxO3a. The findings demonstrated that the wild-type G9a facilitates the nuclear translocation of FoxO3a under H/R condition. In contrast, the K262R-FoxO3a mutation, which lacks the methylation modification, exhibited a marked limitation in nuclear translocation. These results suggest that the methylation of FoxO3a by G9a plays a critical role in its nuclear translocation as well.

In this study, we reported for the first time that TRIM21 is capable of performing the ubiquitination of FoxO3a. TRIM21 is an E3 ubiquitin protein ligase belonging to the ternary motif (TRIM) protein family containing RING structures that is known to act as a major autoantigen in autoimmune diseases and plays a regulatory role in innate immune signaling. TRIM21 Works together with the ubiquitin ligase E2 E1 (UBE2E1), both as an E3 ligase and as a substrate for self-ubiquitinated[49]. Through the screening of numerous TRIM proteins, TRIM21 was identified as the E3 ligase responsible for the ubiquitination of FoxO3a in the context of diabetic RIRI. Subsequently, we conducted a comprehensive investigation into the relationship between G9a methylation and TRIM21-mediated ubiquitination. Our findings provide unequivocal evidence that the ubiquitination of FoxO3a by TRIM21 is contingent upon G9a methylation activity. Mechanistically, G9a facilitates the nuclear translocation of FoxO3a following its methylation at the K262 site, thereby enabling the co-localization of FoxO3a with TRIM21 in the cytoplasm. This interaction subsequently induces the ubiquitination of FoxO3a at the K176 site by TRIM21. Investigating the molecular mechanisms of RIRI under DM is necessary to develop new therapeutic approaches. In this context, we have identified a previously unrecognized function of lysine methylation in the regulation of FoxO3a translocation and degradation mediated by G9a. This interaction attenuate cell pyroptosis induced by IRI both in *vivo* and in vitro under DM. Combine with our prior research, G9a exhibits a dual function in methylating both histone and non-histone proteins during renal ischemia, thereby highlighting its significant regulatory role in renal ischemia injury. Notably, the potential significance of G9a in various cancer types has prompted the development of the specific inhibitor BIX-01294[50]. Administration of this inhibitor has been shown to impede epithelial-to-mesenchymal transition in both cell culture and in *vivo* breast cancer models[51], as well as to decrease proliferation rates in certain leukemia cell lines[52]. In our experiment, BIX-01294 also exhibited a significant protective effect against diabetic RIRI. Furthermore, the enhanced inhibitors UNC0638, BRD4770, UNC0642, and A-366 have now attained cellular toxicity, target specificity, and pharmacokinetic profiles that are appropriate for animal studies[53-56]. Collectively, these findings indicate that G9a inhibitors hold substantial promise for the treatment of diabetic RIRI.

In conclusion, our study has first identified that G9a methylates FoxO3a in a non-histone form, resulting in its translocation and K48-linked ubiquitination mediated by TRIM21, which subsequently leads to the degradation of FoxO3a. This process plays a regulatory role in oxidative stress and the prognosis of diabetic RIRI. These effects are closely associated with the methylation site K262 and the ubiquitination site K176 on FoxO3a. And the development of G9a inhibitors or FoxO3a agonists holds significant promise for the treatment of diabetic RIRI. Hence, our findings provide critical and novel strategies for the treatment of diabetic RIRI.

**Declarations**

**Ethics approval and consent to participate**

All experiments involving animals were conducted according to the ethical policies and procedures approved by the Research Ethics Committee of Renmin Hospital of Wuhan University (Approval no. WDRM20191006).

**Consent for publication**

Not applicable.

**Availability of data and materials**

The datasets during and/or analysed during the current study available from the corresponding author on reasonable request.

**Competing interests**

The authors declare that they have no competing interests.

**Author contributions**

Qingyuan Zheng: Conceptualization, Methodology, Validation, Writing-original draft, Writing-review & editing. Xuke Qin: Conceptualization, Methodology, Investigation, Writing-review & editing. Shiyu Hang: Conceptualization, Methodology, Resources, Writing-review & editing. Zhiwei Yan: Investigation, Resource. Jin Liu: Formal Analysis, Software. Yufeng Xiong: Validation, Visualization. Xiaojie Zhao: Investigation, Resource. Xinmiao Ni: Investigation, software. Haonan Mei: Investigation, software. Jiaxin Wang: Investigation, software. Jun Jian: Software, Validation. Jingsong Wang: visualization. Qianxue Lu: Writing-review & editing. Zhiyuan Chen: Supervision, Funding acquisition. Xiuheng Liu: Project Administration, Writing-review & editing. Shanshan Wan: Data curation, Resource. Hao Liu: Funding acquisition, Writing-review & editing. Lei Wang: Funding acquisition, Conceptualization. All authors read and approved the final manuscript.

**Funding**

This study was supported by National Natural Science Foundation of China (Grants No.82000639 and 82372200) and Natural Science Foundation of Hubei Province (No.2025AFB803).

**Acknowledgements**

The authors thank Prof. Mingxia Fan and the staff from the Animal Experiment Center at the Renmin Hospital of Wuhan University for their technical support.

**Reference**

1. Ronco, C., R. Bellomo, and J.A. Kellum, *Acute kidney injury.* Lancet, 2019. **394**(10212): p. 1949-1964.

2. See, E.J., et al., *Long-term risk of adverse outcomes after acute kidney injury: a systematic review and meta-analysis of cohort studies using consensus definitions of exposure.* Kidney Int, 2019. **95**(1): p. 160-172.

3. Chertow, G.M., et al., *Acute kidney injury, mortality, length of stay, and costs in hospitalized patients.* J Am Soc Nephrol, 2005. **16**(11): p. 3365-70.

4. Beker, B.M., et al., *Novel acute kidney injury biomarkers: their characteristics, utility and concerns.* Int Urol Nephrol, 2018. **50**(4): p. 705-713.

5. Yang, Y.Y., et al., *Diabetes aggravates renal ischemia-reperfusion injury by repressing mitochondrial function and PINK1/Parkin-mediated mitophagy.* Am J Physiol Renal Physiol, 2019. **317**(4): p. F852-F864.

6. de Ponte, M.C., et al., *Early type 1 diabetes aggravates renal ischemia/reperfusion-induced acute kidney injury.* Sci Rep, 2021. **11**(1): p. 19028.

7. Olsen, J.V. and M. Mann, *Status of large-scale analysis of post-translational modifications by mass spectrometry.* Mol Cell Proteomics, 2013. **12**(12): p. 3444-52.

8. Mertins, P., et al., *Integrated proteomic analysis of post-translational modifications by serial enrichment.* Nat Methods, 2013. **10**(7): p. 634-7.

9. Murn, J. and Y. Shi, *The winding path of protein methylation research: milestones and new frontiers.* Nat Rev Mol Cell Biol, 2017. **18**(8): p. 517-527.

10. Aziz, N., et al., *Tumor-suppressive functions of protein lysine methyltransferases.* Exp Mol Med, 2023. **55**(12): p. 2475-2497.

11. Cornett, E.M., et al., *Lysine Methylation Regulators Moonlighting outside the Epigenome.* Mol Cell, 2019. **75**(6): p. 1092-1101.

12. Jakobsson, M.E., et al., *Hsp70 (HSPA1) Lysine Methylation Status as a Potential Prognostic Factor in Metastatic High-Grade Serous Carcinoma.* PLoS One, 2015. **10**(10): p. e0140168.

13. Cong, R., et al., *macroH2A1 histone variant represses rDNA transcription.* Nucleic Acids Res, 2014. **42**(1): p. 181-92.

14. Padeken, J., S.P. Methot, and S.M. Gasser, *Establishment of H3K9-methylated heterochromatin and its functions in tissue differentiation and maintenance.* Nat Rev Mol Cell Biol, 2022. **23**(9): p. 623-640.

15. Zhang, Y., et al., *Histone methyltransferase G9a protects against acute liver injury through GSTP1.* Cell Death Differ, 2020. **27**(4): p. 1243-1258.

16. Xue, W., et al., *Histone methyltransferase G9a modulates hepatic insulin signaling via regulating HMGA1.* Biochim Biophys Acta Mol Basis Dis, 2018. **1864**(2): p. 338-346.

17. Zhang, W., et al., *Muscular G9a Regulates Muscle-Liver-Fat Axis by Musclin Under Overnutrition in Female Mice.* Diabetes, 2020. **69**(12): p. 2642-2654.

18. Irifuku, T., et al., *Inhibition of H3K9 histone methyltransferase G9a attenuates renal fibrosis and retains klotho expression.* Kidney Int, 2016. **89**(1): p. 147-57.

19. Sung, P., et al., *Inhibition of histone methyltransferase G9a effectively protected the kidney against ischemia-reperfusion injury.* Am J Transl Res, 2022. **14**(6): p. 3683-3697.

20. Liu, H., et al., *The H3K9 histone methyltransferase G9a modulates renal ischemia reperfusion injury by targeting Sirt1.* Free Radic Biol Med, 2021. **172**: p. 123-135.

21. Clark, K.L., et al., *Co-crystal structure of the HNF-3/fork head DNA-recognition motif resembles histone H5.* Nature, 1993. **364**(6436): p. 412-20.

22. Huang, H. and D.J. Tindall, *Dynamic FoxO transcription factors.* J Cell Sci, 2007. **120**(Pt 15): p. 2479-87.

23. Zhang, X., et al., *Akt, FoxO and regulation of apoptosis.* Biochim Biophys Acta, 2011. **1813**(11): p. 1978-86.

24. Fu, Z. and D.J. Tindall, *FOXOs, cancer and regulation of apoptosis.* Oncogene, 2008. **27**(16): p. 2312-9.

25. Kant, S., et al., *Neural JNK3 regulates blood flow recovery after hindlimb ischemia in mice via an Egr1/Creb1 axis.* Nat Commun, 2019. **10**(1): p. 4223.

26. Wu, X., et al., *NLRP3 inﬂammasome mediates chronic intermittent hypoxia-induced renal injury implication of the microRNA-155/FOXO3a signaling pathway.* J Cell Physiol, 2018. **233**(12): p. 9404-9415.

27. Das, M., et al., *Caveolin induces cardioprotection through epigenetic regulation.* J Cell Mol Med, 2012. **16**(4): p. 888-95.

28. Liu, H., et al., *Inhibition of Brd4 alleviates renal ischemia/reperfusion injury-induced apoptosis and endoplasmic reticulum stress by blocking FoxO4-mediated oxidative stress.* Redox Biol, 2019. **24**: p. 101195.

29. Liu, H., et al., *Enhancer of zeste homolog 2 modulates oxidative stress-mediated pyroptosis in vitro and in a mouse kidney ischemia-reperfusion injury model.* FASEB J, 2020. **34**(1): p. 835-852.

30. Jablonski, P., et al., *An experimental model for assessment of renal recovery from warm ischemia.* Transplantation, 1983. **35**(3): p. 198-204.

31. Black, J.C., C. Van Rechem, and J.R. Whetstine, *Histone lysine methylation dynamics: establishment, regulation, and biological impact.* Mol Cell, 2012. **48**(4): p. 491-507.

32. Xu, J. and S. Richard, *Cellular pathways influenced by protein arginine methylation: Implications for cancer.* Mol Cell, 2021. **81**(21): p. 4357-4368.

33. Huang, J., et al., *G9a and Glp methylate lysine 373 in the tumor suppressor p53.* J Biol Chem, 2010. **285**(13): p. 9636-9641.

34. Chuikov, S., et al., *Regulation of p53 activity through lysine methylation.* Nature, 2004. **432**(7015): p. 353-60.

35. Huang, J., et al., *Repression of p53 activity by Smyd2-mediated methylation.* Nature, 2006. **444**(7119): p. 629-32.

36. Shi, X., et al., *Modulation of p53 function by SET8-mediated methylation at lysine 382.* Mol Cell, 2007. **27**(4): p. 636-46.

37. Barbier-Torres, L., et al., *Depletion of mitochondrial methionine adenosyltransferase alpha1 triggers mitochondrial dysfunction in alcohol-associated liver disease.* Nat Commun, 2022. **13**(1): p. 557.

38. Ambrosini, S., et al., *Methylation of the Hippo effector YAP by the methyltransferase SETD7 drives myocardial ischaemic injury: a translational study.* Cardiovasc Res, 2023. **118**(17): p. 3374-3385.

39. Hamamoto, R., V. Saloura, and Y. Nakamura, *Critical roles of non-histone protein lysine methylation in human tumorigenesis.* Nat Rev Cancer, 2015. **15**(2): p. 110-24.

40. Pefanis, A., et al., *Regulated necrosis in kidney ischemia-reperfusion injury.* Kidney Int, 2019. **96**(2): p. 291-301.

41. Zhao, Z., et al., *XJB-5-131 inhibited ferroptosis in tubular epithelial cells after ischemia-reperfusion injury.* Cell Death Dis, 2020. **11**(8): p. 629.

42. Ma, N., et al., *CHOP-mediated Gasdermin E expression promotes pyroptosis, inflammation, and mitochondrial damage in renal ischemia-reperfusion injury.* Cell Death Dis, 2024. **15**(2): p. 163.

43. Spel, L. and F. Martinon, *Inflammasomes contributing to inflammation in arthritis.* Immunol Rev, 2020. **294**(1): p. 48-62.

44. Fu, J. and H. Wu, *Structural Mechanisms of NLRP3 Inflammasome Assembly and Activation.* Annu Rev Immunol, 2023. **41**: p. 301-316.

45. Mangan, M.S.J., et al., *Targeting the NLRP3 inflammasome in inflammatory diseases.* Nat Rev Drug Discov, 2018. **17**(8): p. 588-606.

46. Samir, P., et al., *DDX3X acts as a live-or-die checkpoint in stressed cells by regulating NLRP3 inflammasome.* Nature, 2019. **573**(7775): p. 590-594.

47. Krimpenfort, P. and A. Berns, *Rejuvenation by Therapeutic Elimination of Senescent Cells.* Cell, 2017. **169**(1): p. 3-5.

48. Iwai, K., *LUBAC-mediated linear ubiquitination: a crucial regulator of immune signaling.* Proceedings of the Japan Academy, Series B, 2021. **97**(3): p. 120-133.

49. Anandapadamanaban, M., et al., *E3 ubiquitin-protein ligase TRIM21-mediated lysine capture by UBE2E1 reveals substrate-targeting mode of a ubiquitin-conjugating E2.* J Biol Chem, 2019. **294**(30): p. 11404-11419.

50. Kubicek, S., et al., *Reversal of H3K9me2 by a small-molecule inhibitor for the G9a histone methyltransferase.* Mol Cell, 2007. **25**(3): p. 473-81.

51. Dong, C., et al., *G9a interacts with Snail and is critical for Snail-mediated E-cadherin repression in human breast cancer.* J Clin Invest, 2012. **122**(4): p. 1469-86.

52. Savickiene, J., et al., *Euchromatic histone methyltransferase 2 inhibitor, BIX-01294, sensitizes human promyelocytic leukemia HL-60 and NB4 cells to growth inhibition and differentiation.* Leuk Res, 2014. **38**(7): p. 822-9.

53. Vedadi, M., et al., *A chemical probe selectively inhibits G9a and GLP methyltransferase activity in cells.* Nat Chem Biol, 2011. **7**(8): p. 566-74.

54. Yuan, Y., et al., *A small-molecule probe of the histone methyltransferase G9a induces cellular senescence in pancreatic adenocarcinoma.* ACS Chem Biol, 2012. **7**(7): p. 1152-7.

55. Liu, F., et al., *Discovery of an in vivo chemical probe of the lysine methyltransferases G9a and GLP.* J Med Chem, 2013. **56**(21): p. 8931-42.

56. Sweis, R.F., et al., *Discovery and development of potent and selective inhibitors of histone methyltransferase g9a.* ACS Med Chem Lett, 2014. **5**(2): p. 205-9.

**Supplementary Material**


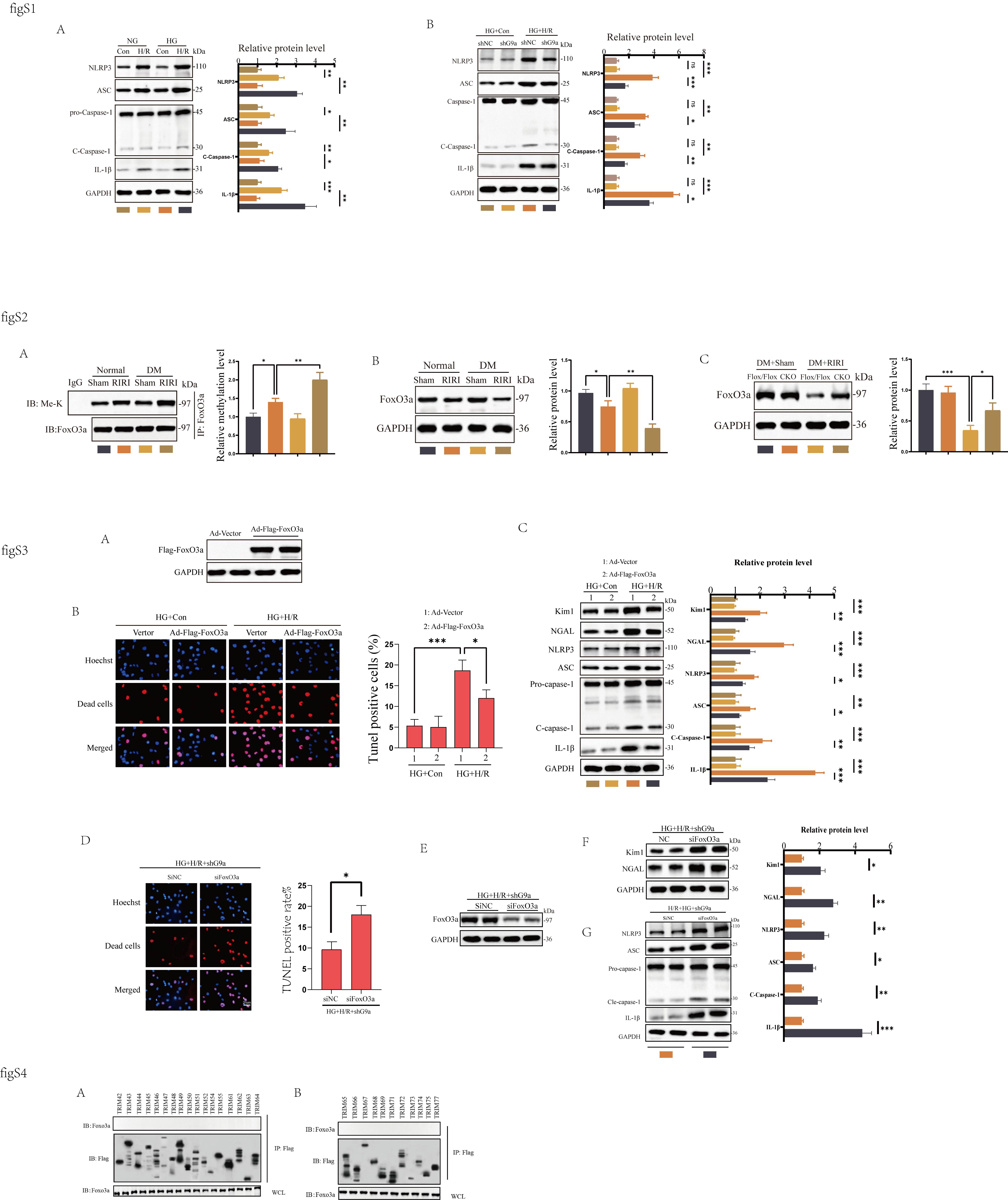


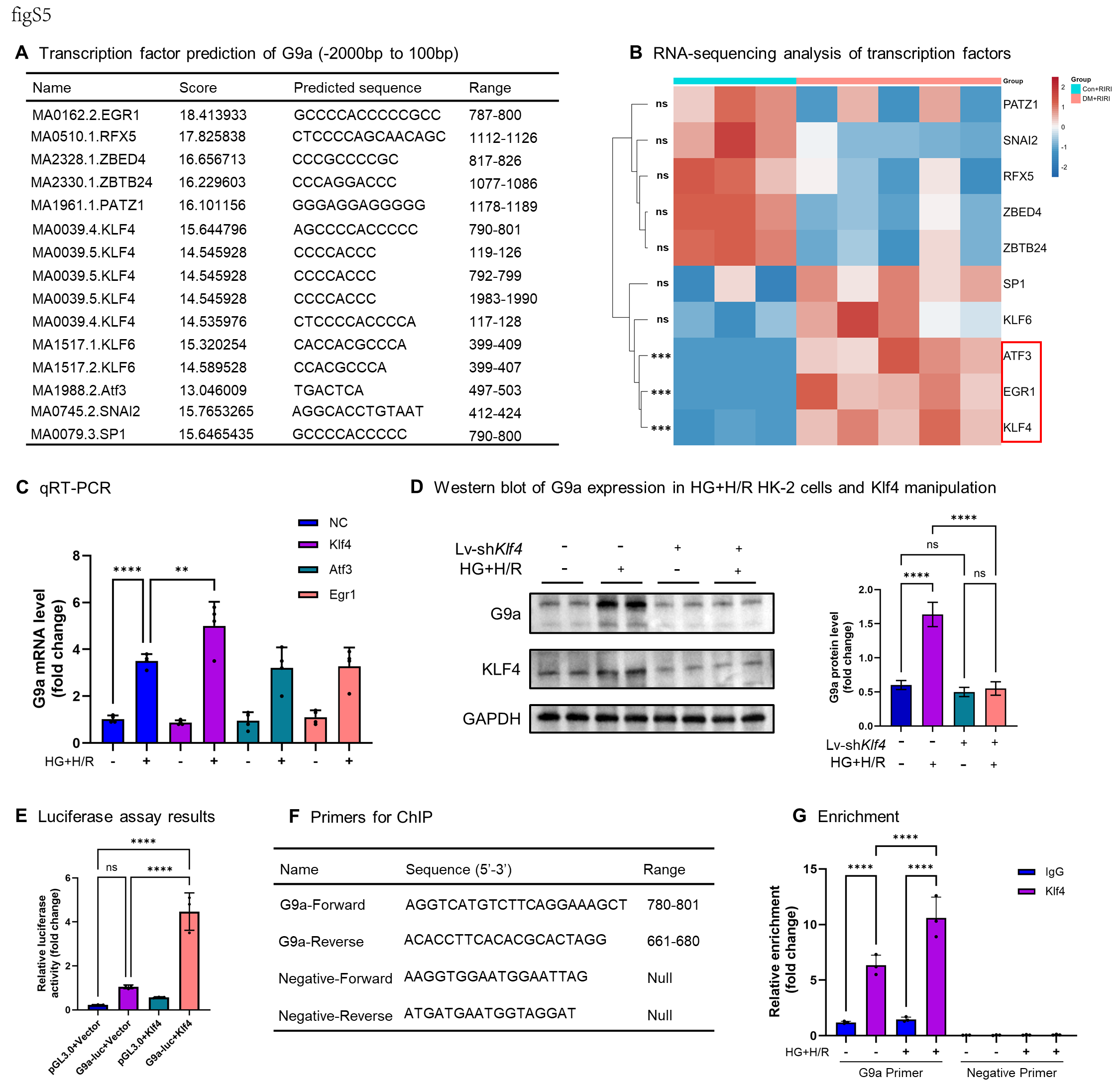

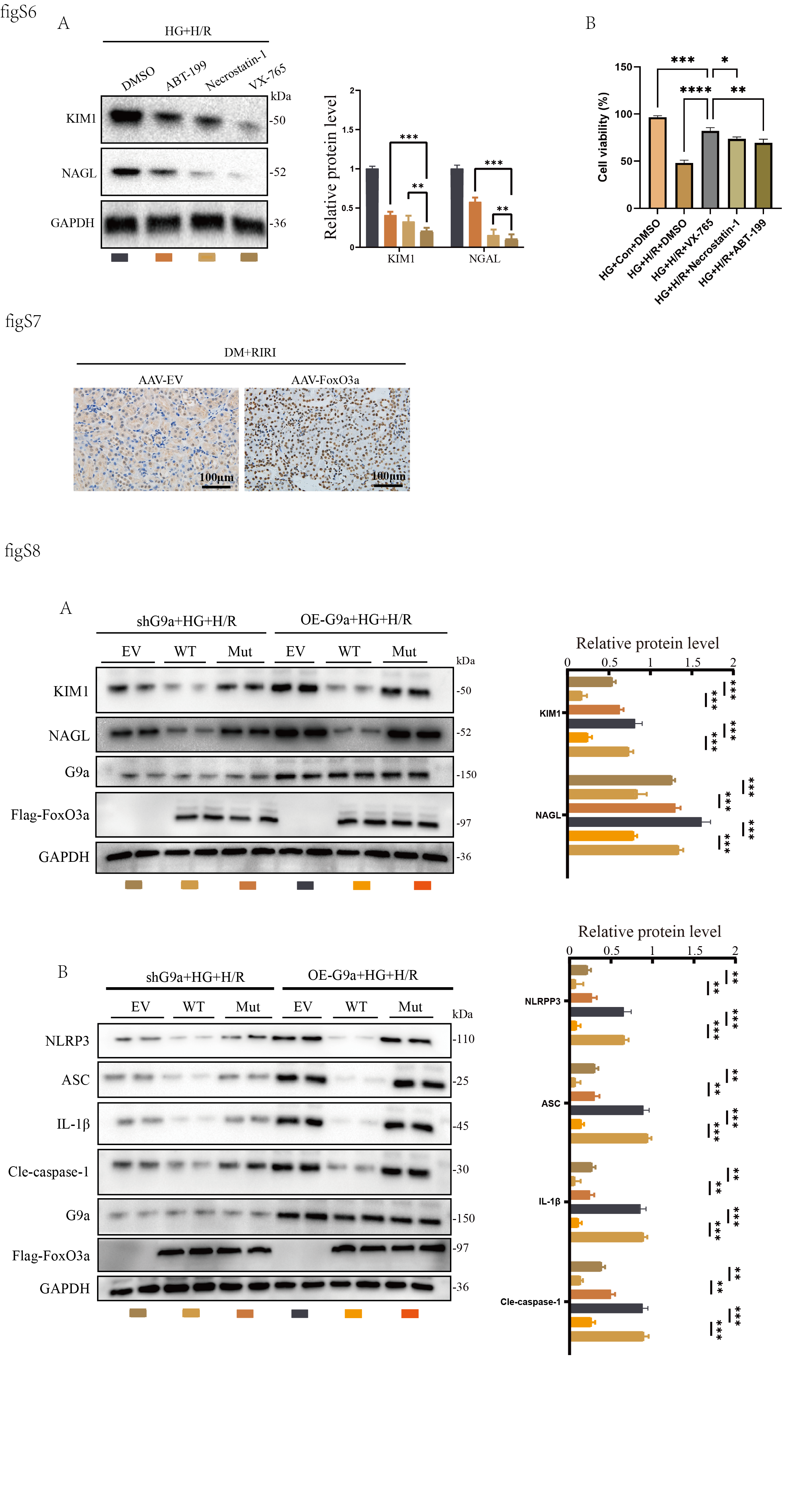


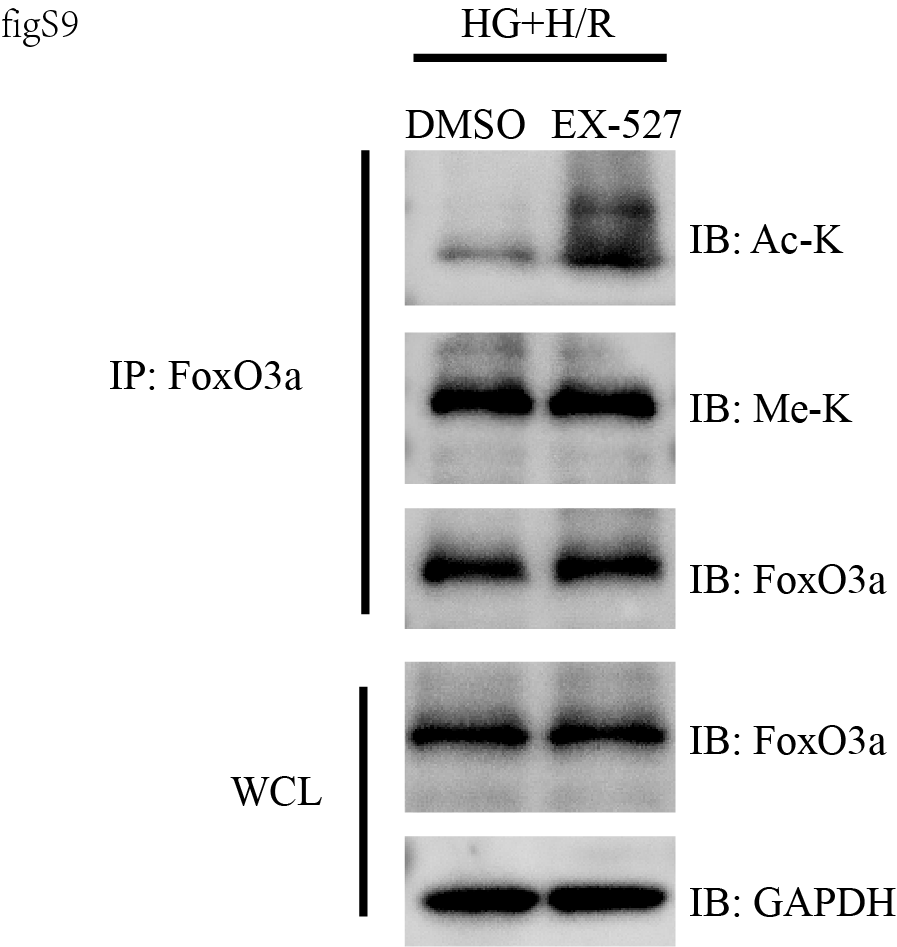

Supplement: Multimedia component 1 [file mmc1.docx]
